# Supplementary material for: Insecticides may facilitate the escape of weeds from biological control
Source: PeerJ. 2025 Jan 13;13:e18597. doi: 10.7717/peerj.18597 (PMC11737330; doi:10.7717/peerj.18597)
Supplement: Supplemental Information 1 [file peerj-13-18597-s001.docx]

**.Supplementary material**

Manuscript Title: Insecticides may facilitate the escape of weeds from biological control

Authors: Elizabeth K. Rowen^a,b^, Kirsten A. Pearsons^b^, Richard G. Smith^c^, Kyle Wickings^d^, John F. Tooker^b^

**Table**

**Table S1:** Planting and field management for each field 2017-2019

| **Year** | **Field** | **Cover crop species** | **Cover crop planting date** | **Cover planting rate** | **Cover termination date**  **(Herbicide Product)** | **Crop** | **Seed variety** | **Cash crop planting date** | **Planting rate (seeds/acre)** | **Seed Coat treatment (product, mg/seed)** | **IPM treatment** | **Herbicide application date during crop growth (Product)** |
| --- | --- | --- | --- | --- | --- | --- | --- | --- | --- | --- | --- | --- |
| **2017** | North | *Avena sativa* | 11-Apr-2017 | 2-3 bu/acre (~ 960K– 1440 K seeds/acre) | 24-May-2017 (Roundup PowerMax [22 fl oz/ac]) | Soy | TS2849R2S (Local Seed Co., Jersey Shore, PA) | 2-Jun-2017 | 180,000 | Trius Elite (imidacloprid, 0.125) |  | 30-June-2017 (Accent) |
|  | South | *Avena sativa* | 11-Apr-2017 | 2-3 bu/acre (~ 960K– 1440 K seeds/acre) | 4-Jun-2017 (Impact, Accent, Banvil, and Degree Extra) | Corn | MC5250 (Masters Choice, Anna, IL) | 19-May-2017 | 30,000 | CruiserMaxx® 250 + Vibrance® (thiamethoxam, 0.250) |  | 3-July-2017 (Round-Up) |
| **2018** | North | *Secale cereale* | 12-Nov-2017 | 50 lbs/acre (~1 million seeds/acre | 21-May-2018 (Roundup PowerMax [22 fl oz/ac]) | Corn | TA477-18 (Local Seed Co. Jersey Shore PA) | 30-May-2018 | 32,000 | CruiserMaxx® 250 (thiamethoxam, 0.250) | In furrow granular application of Force 3G (8 g a.i./ ha) | 26-Jun-2018 (Round-Up PowerMax2) |
|  | South | *Secale cereale* | 22-Nov-2017 | 50 lbs/acre (~1 million seeds/acre | 23-May-2018 (Roundup PowerMax ([22 fl oz/ac]) | Soy | TS2849R2S (Local Seed Co. Jersey Shore PA) | 14-June-2018 and 26-June-2018 | 178,000 | Trius Elite™ (imidacloprid, 0.125) |  | 29-Jun-2018 (Round-Up PowerMax) |
| **2019** | North | *Avena sativa* | 29-Mar-2019 | 2-3 bu/acre (~ 960K– 1440 K seeds/acre) | 4-Jun-2019 (Roundup PowerMax [28 oz/ac]) | Soy | LS2847R2S (Local Seed Co., Jersey Shore, PA) | 22-May-2019 | 180,000 | Radius Premium (thiamethoxam, 0.0756) |  |  |
|  | South | *Avena sativa* | 29-Mar-2019 | 2-3 bu/acre (~ 960K– 1440 K seeds/acre) | 22-May-2019 (Roundup PowerMax (28 oz/ac), Acuron (3 qt/ac)) | Corn | ZS9725 GT (Local Seed Co., Jersey Shore, PA) | 17-May-2019 | 30,000 | Radius 500 (clothianidin, 0.500) |  |  |

**Figures:**

**
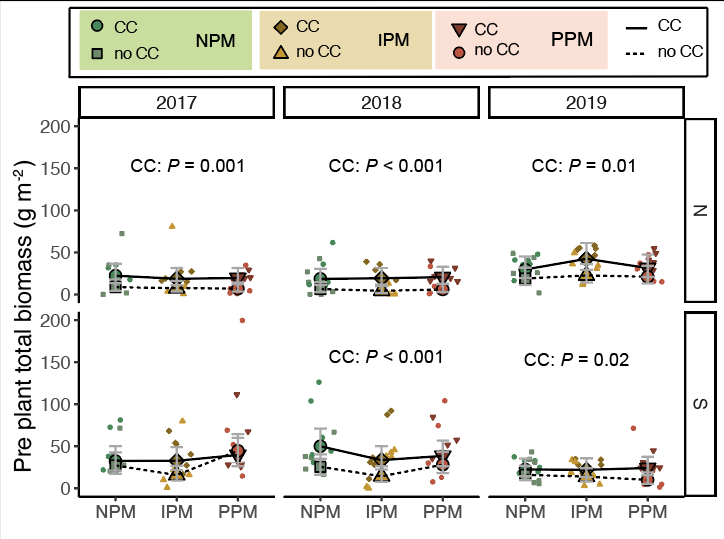
**

**Figure S1**: Estimated marginal means (95% confidence intervals [CIs]) of total pre-plant biomass (g m^-2^) before planting in each field and year. GLMM (lognormal distribution), *᙭²* = 159, *df* = 23, *P* < 0.001, *N* = 209. Significance of cover crop treatments (CC) included in panels where GLM indicated cover crops had a significant effect by year/field slicing. Means for treatments with cover crops indicated with a solid line, means without cover crop indicated by a dashed line. Raw data shown as open small shapes behind means and CIs.


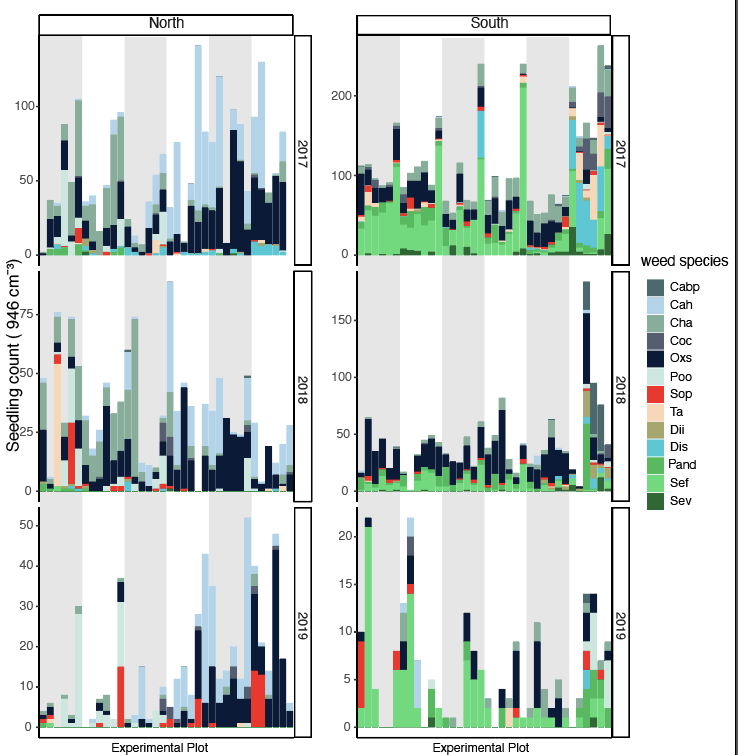


**Figure S2**: Weed community in the weed seed bank. Stacked bars are colored by weed species with > 20 individuals recovered over three years. Grey stripes show one direction of blocking in Latin-square experimental design. Species abbreviations: Cabo = *Capsella bursa-pastoris* (Shepard’s purse), Cah = *Cardamine hirsuta* (hairy bittercress), Cha = *Chenopodium album* (lambsquarter), Coc = *Erigeron canadensis* (marestail), Osx = *Oxalis stricta* (yellow woodsorrel) Poo = *Portulaca oleracea* (purslane), Sop = *Solanum ptychanthum* (eastern black nightshade),Ta = *Taraxacum* spp. (dandelion), Dii = *Digitaria ischaemum* (smooth crabgrass), Pand = *Panicum dichotomiflorum* (fall panicum), Sef = *Setaria faberi* (giant foxtail), Sev = *Setaria viridis* (green foxtail).

**
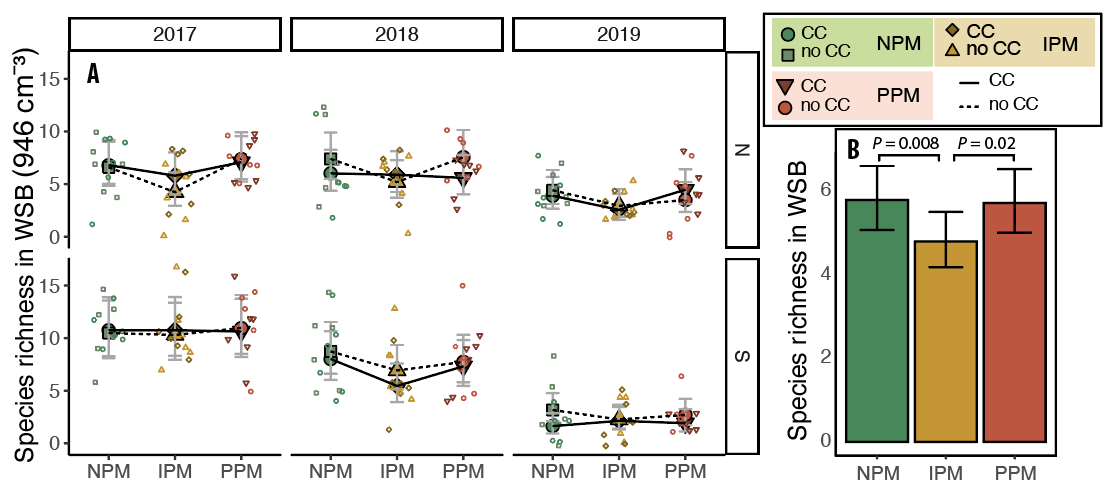
**

**Figure S3**: Estimated marginal means (95% confidence intervals [CIs]) of species richness in the weed-seed bank before planting A) in each field and year, and B) for all years and fields combined. In A) means for treatments with cover crops indicated with a solid line, means without cover crop indicated by a dashed line. Raw data shown as open small shapes behind means and CIs. GLMM (Poisson distribution), *᙭²* = 217, *df* = 8, *P* < 0.0001, *N* = 216. Significance of PM treatments included in panels where GLMM indicated PM had a significant effect, and the *P* values for pairwise comparisons are given.


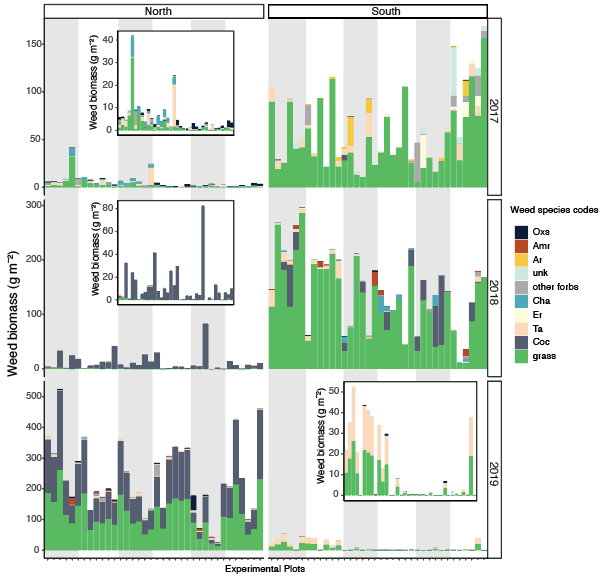


**Figure S4**: Weed community in August in each experimental plot. Stacked bars are colored by weed species with > 50 g biomass collected over three years. All other weeds are grouped in “other forbs”. Grey stripes show one direction of blocking in Latin-square experimental design. Insets show weed biomass at finer resolution for field years with lower weed biomass. Species abbreviations: Amr = *Amaranthus retroflexus* (redroot pigweed), Ar = *Arcticum* spp. (burdock), Cha = *Chenopodium album* (lambsquarter), Coc = *Conyza canadensis* (marestail), Er = *Erigeron* spp., Ta = *Taraxacum* spp. (dandelion), unk = unknown sp.

**
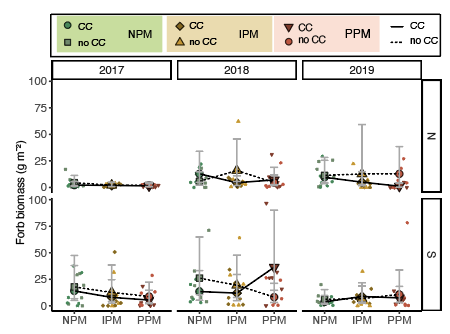
**

**Figure S5**: Estimated marginal means (95% confidence intervals [CIs]) of forb biomass in August for each field and year. Model information: GLMM (Poisson distribution), *᙭²* = 43, *df* = 8, *P* < 0.001, *N* = 216. Means for treatments with cover crops indicated with a solid line, means without cover crop indicated by a dashed line. Raw data shown as open small shapes behind means and CIs.


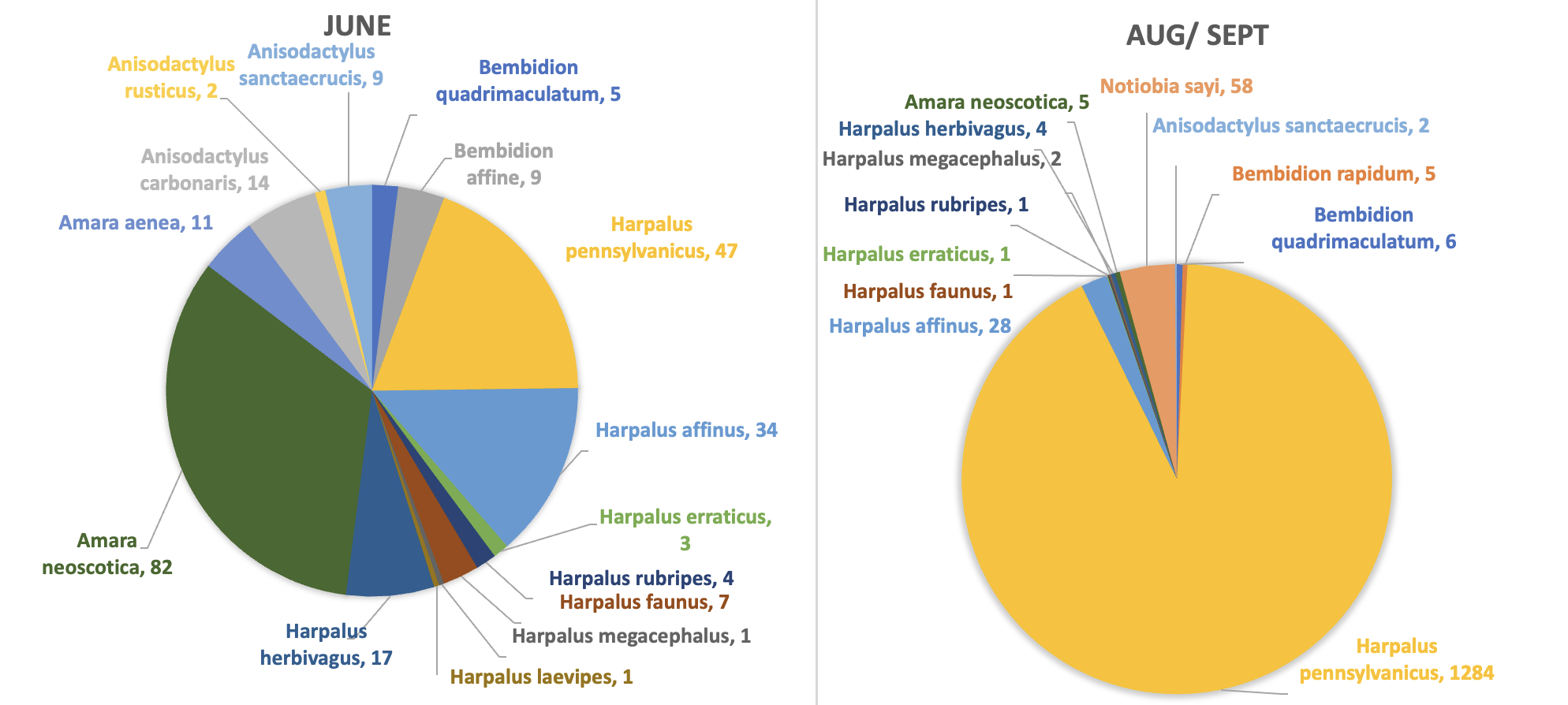


**Figure S6:** Pie charts of community composition of all granivorous carabids collected across all fields and years in June and August/September. Sums of number of individuals from species are reported with the species name.


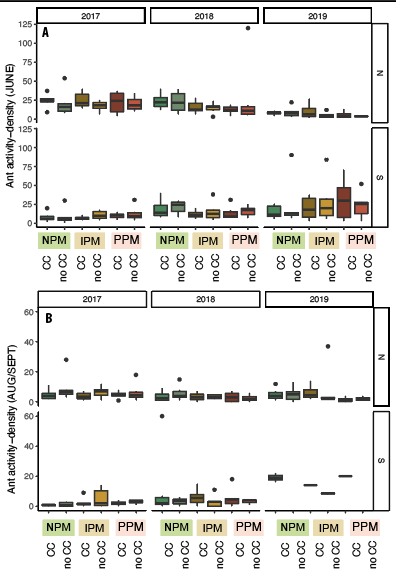


**Figure S7**: Boxplots of ant activity-density for in A) June/July and B) Aug/Sept for each field and year. Model information: GLMM (negative binomial distribution), *᙭²* = 256, *df* = 11, *P* < 0.001, *N* = 395.
