# Supplementary material for: Insecticides may facilitate the escape of weeds from biological control
Source: PeerJ. 2025 Jan 13;13:e18597. doi: 10.7717/peerj.18597 (PMC11737330; doi:10.7717/peerj.18597)
Supplement: Supplemental Information 3 [file peerj-13-18597-s003.html]

Pre-plant weed and cover crop biomass Rowen et al. 2024


# Pre-plant weed and cover crop biomass Rowen et al. 2024

#### Elizabeth Rowen

#### 5/6/2024

# Context for this document

This document includes the code for Rowen et al. submitted to Field
Crops Research in 2024 titled ” Insecticides may facilitate the escape
of weeds from biological control”

**This includes the code and statistical output for the
Pre-plant cover data. The code covers Results section 3.1, Fig 1, and
Fig S1.**

**Abstract** 1. CONTEXT: Preventative pesticide seed
treatments (hereafter preventative pest management or PPM) are common in
large acreage row crops such as corn and soybean, and often include
active ingredients of both fungicides and neonicotinoid insecticides.
While PPM is intended to protect crops from soil-borne pathogens and
early season insect pests, these seed treatments may have detrimental
effects on biological control of weed seeds by insects.  
2. RESEARCH QUESTION: Here, in two 3-year corn-soy rotations in
Pennsylvania USA, we investigated a PPM approach to insect management
compared to an integrated pest management approach (IPM) and a “no
(insect) pest management” (NPM) control. This was crossed with a grass
cover crop treatment to see if this conservation practice can help
recover some of the ecosystem services affected by chemical pest
management practices. We hypothesized that PPM and IPM approaches would
release weed seeds from biological control by insects, resulting in
higher weed pressure, but cover crops would increase biological
control.  
3. METHODS: We measured the effect of these treatments on granivorous
insect activity-density, weed-seed predation, the weed-seed bank, and
mid-season weed biomass.  
4. RESULTS: We found that, contrary to our hypothesis, planting a cover
crop decreased carabid activity-density, but did not have consistent
differences in weed-seed predation. Pest management and cover crop
treatments also had inconsistent effects on the weed-seed bank and
mid-season weed biomass, but insecticide use did affect the biomass of
glyphosate-resistant marestail (*Erigeron canadensis* L.). At the
end of the trial, glyphosate-resistant marestail was more abundant in
IPM and PPM plots without a cover crop.  
5. IMPLICATIONS: Our results suggest that reducing insecticide use may
be important when combating herbicide-resistant weeds. As the adoption
conservation agriculture, including the use of no-till and cover crops,
grows, so does the use of neonicotinoid seed treatments and herbicides.
Planting cover crops and/or avoiding the use of insecticides may combat
these problematic weeds.;

### Set-up

Import libraries

```
# importing and formatting data 
library(readxl)
library(plyr)
library(flextable)
library(reshape2)

# creating figures 

library(ggplot2)

# Running models 
library(car)
library(glmmTMB)
```

```
## Warning in check_dep_version(dep_pkg = "TMB"): package version mismatch: 
## glmmTMB was built with TMB package version 1.9.15
## Current TMB package version is 1.9.14
## Please re-install glmmTMB from source or restore original 'TMB' package (see '?reinstalling' for more information)
```

```
library(DHARMa)
library(vegan)
library(pscl)
#library(VGAM)
library(performance)
library(corrplot)
library(emmeans)
```

Set up colors for graphs

```
colors = c("seagreen","darkseagreen4","darkgoldenrod4", "darkgoldenrod3","tomato4","tomato3") 
colors.treat = c("seagreen","darkgoldenrod3","tomato3") # only for insecticide treatments 
colors.2017 = c("#085C3D","seagreen","#FC9078","#FFE2DB") # for 2017 when IPM had not yet been implemented 
# c("seagreen","darkseagreen4","tomato3","tomato4")
```

## Weed and total biomass in May/June

Import data

```
preplant.cover.data <- read_excel("~/Dropbox/1PhD/Smith & Wickings Field Experiments (AKA smickings)/Manuscripts in progress/Weeds/PeerJ/Data&Stats/1_PreplantCover_Apr2021.xlsx")


# format treatment,cover crop for each plot ID
preplant.cover.data=mutate(preplant.cover.data,Treatment=ifelse(Plot_ID=='105N' | Plot_ID=='106N'| Plot_ID=='203N'| Plot_ID=='204N'|Plot_ID=='305N'| Plot_ID=='306N'| 
                                                                  Plot_ID=='401N'|Plot_ID=='402N'| Plot_ID=='501N'| Plot_ID=='504N'| Plot_ID=='602N'| Plot_ID=='603N'| 
                                                                  Plot_ID=='102S'| Plot_ID=='106S'| Plot_ID=='201S'| Plot_ID=='204S'| Plot_ID=='303S'| Plot_ID=='305S'| 
                                                                  Plot_ID=='402S'| Plot_ID=='406S'| Plot_ID=='501S'| Plot_ID=='504S'| Plot_ID=='603S'|Plot_ID=='605S','1NPM', 
                                                                ifelse(Plot_ID=='101N' | Plot_ID=='104N'| Plot_ID=='202N'| Plot_ID=='205N'| Plot_ID=='301N'| Plot_ID=='303N'| 
                                                                         Plot_ID=='405N'| Plot_ID=='406N'| Plot_ID=='502N'| Plot_ID=='503N'| Plot_ID=='604N'| Plot_ID=='606N'| 
                                                                         Plot_ID=='104S'| Plot_ID=='105S' | Plot_ID=='202S'| Plot_ID=='203S'| Plot_ID=='301S'| 
                                                                         Plot_ID=='306S'| Plot_ID=='404S'| Plot_ID=='405S'| Plot_ID=='502S'| Plot_ID=='503S'| Plot_ID=='601S'| 
                                                                         Plot_ID=='606S','2IPM','3PPM')))


preplant.cover.data=mutate(preplant.cover.data,cover_crop=ifelse(Plot_ID=='101N' | Plot_ID=='102N'| Plot_ID=='106N'| Plot_ID=='203N'|Plot_ID=='205N'| Plot_ID=='206N'|
                                                                   Plot_ID=='303N'| Plot_ID=='304N'| Plot_ID=='305N'| Plot_ID=='401N'| Plot_ID=='403N'| Plot_ID=='406N'| 
                                                                   Plot_ID=='502N'| Plot_ID=='504N'|Plot_ID=='505N'| Plot_ID=='601N'| Plot_ID=='602N'| Plot_ID=='604N'| 
                                                                   Plot_ID=='102S'| Plot_ID=='103S'| Plot_ID=='105S'| Plot_ID=='202S'| Plot_ID=='204S'| Plot_ID=='206S'| 
                                                                   Plot_ID=='303S'| Plot_ID=='304S'| Plot_ID=='306S'| Plot_ID=='401S'|  Plot_ID=='404S'| Plot_ID=='406S'| 
                                                                   Plot_ID=='501S'| Plot_ID=='503S'| Plot_ID=='505S'| Plot_ID=='601S'| Plot_ID=='602S'|Plot_ID=='605S','cover', 'fallow'))
preplant.cover.data=mutate(preplant.cover.data,Treated=ifelse(Year == '2017' &Treatment=='2IPM'|Treatment== '1NPM','1NPM',Treatment))
preplant.cover.data$Block <- as.factor(preplant.cover.data$Block)
preplant.cover.data$Plot <- as.factor(preplant.cover.data$Plot)
preplant.cover.data$Year <- as.factor(preplant.cover.data$Year)
preplant.cover.data$Total.biomass_g <- preplant.cover.data$`Total biomass_g`
```

To analyze data, we used a 4-way interaction model with Treatment,
cover crop, year and field and random effects: Plot ID (experimental
unit), and blocking factors Block (column) and Plot (row in Latin-square
design). ### Weed stats for each field year (Table 1)

```
total.biomass.summary <- ddply(preplant.cover.data, .(Year, Field), summarize, mean.biomass.biomass = mean (Total.biomass_g,na.rm=T), N = sum(!is.na(Total.biomass_g)),sd=sd(Total.biomass_g,na.rm=T),se=sd/sqrt(N));total.biomass.summary
```

```
##   Year Field mean.biomass.biomass  N       sd       se
## 1 2017     N             15.23212 33 13.56819 2.361920
## 2 2017     S             34.16242 33 28.11347 4.893928
## 3 2018     N             12.78972 36 11.29875 1.883124
## 4 2018     S             34.72500 36 22.36183 3.726972
## 5 2019     N             26.45611 36 11.01884 1.836473
## 6 2019     S             18.01086 35 10.92312 1.846344
```

```
weed.summary <- ddply(preplant.cover.data, .(Year, Field), summarize, mean.weed.biomass = mean (Weeds_g,na.rm=T), N = sum(!is.na(Weeds_g)),sd=sd(Weeds_g,na.rm=T),se=sd/sqrt(N));weed.summary
```

```
##   Year Field mean.weed.biomass  N        sd       se
## 1 2017     N          8.600606 33 14.330445 2.494610
## 2 2017     S         30.282059 34 28.411388 4.872513
## 3 2018     N          7.826562 32  9.492108 1.677983
## 4 2018     S         28.625000 36 22.291405 3.715234
## 5 2019     N         15.423429 35 10.871651 1.837644
## 6 2019     S         11.638235 34 10.991936 1.885101
```

### Weed biomass in May/June

```
modelPrePlantWeeds.full <- glmmTMB(Weeds_g/0.75~Treatment*cover_crop*Year*Field+(1|Plot_ID:Field)+(1|Block:Field)+(1|Plot:Field),family="lognormal",data=subset(preplant.cover.data))
# using full model for graphing, but will report a more parsimonious model 
#summaryOutput <- summary(modelPrePlantWeeds.full)
#anovaOutput <- Anova(modelPrePlantWeeds.full)
#r2Output <- r2_nakagawa(modelPrePlantWeeds.full)

simres <- simulateResiduals(modelPrePlantWeeds.full) 
plot(simres)
```

```
x=emmeans(modelPrePlantWeeds.full, ~cover_crop:Treatment:Field:Year,type="response")

weed.preplant.table = as.data.frame(x)

plot(check_collinearity(modelPrePlantWeeds.full))
```

Reducing non-significant interactions. The results of this model are
reported in the text. Note: some interactions are not significant but
they help with model fit, and are thus retained.

```
modelPrePlantWeeds <- glmmTMB(Weeds_g/0.75~Treatment+cover_crop*Field*Year+(1|Plot_ID:Field)+(1|Block:Field)+(1|Plot:Field),family="lognormal",data=subset(preplant.cover.data)) 
summaryOutput <- summary(modelPrePlantWeeds)
anovaOutput <- Anova(modelPrePlantWeeds,type=3)
r2Output <- r2_nakagawa(modelPrePlantWeeds)

check_collinearity(modelPrePlantWeeds)
```

```
## # Check for Multicollinearity
## 
## Low Correlation
## 
##       Term  VIF     VIF 95% CI Increased SE Tolerance Tolerance 95% CI
##  Treatment 1.08 [ 1.01,  1.49]         1.04      0.92     [0.67, 0.99]
##      Field 2.58 [ 2.13,  3.20]         1.61      0.39     [0.31, 0.47]
## 
## Moderate Correlation
## 
##              Term  VIF     VIF 95% CI Increased SE Tolerance Tolerance 95% CI
##        cover_crop 8.94 [ 7.08, 11.37]         2.99      0.11     [0.09, 0.14]
##  cover_crop:Field 8.66 [ 6.86, 11.01]         2.94      0.12     [0.09, 0.15]
## 
## High Correlation
## 
##                   Term   VIF     VIF 95% CI Increased SE Tolerance
##                   Year 56.93 [44.42, 73.06]         7.55      0.02
##        cover_crop:Year 71.82 [55.99, 92.19]         8.47      0.01
##             Field:Year 55.83 [43.56, 71.64]         7.47      0.02
##  cover_crop:Field:Year 59.64 [46.52, 76.54]         7.72      0.02
##  Tolerance 95% CI
##      [0.01, 0.02]
##      [0.01, 0.02]
##      [0.01, 0.02]
##      [0.01, 0.02]
```

```
# test the fit of fixed factors 
modelPrePlantWeeds.null <- glmmTMB(Weeds_g/0.75~1+(1|Plot_ID:Field)+(1|Block:Field)+(1|Plot:Field),family=nbinom2,data=subset(preplant.cover.data))
```

```
## Warning in glmmTMB(Weeds_g/0.75 ~ 1 + (1 | Plot_ID:Field) + (1 | Block:Field) +
## : non-integer counts in a nbinom2 model
```

```
model_null_comp <- anova(modelPrePlantWeeds,modelPrePlantWeeds.null)


simres <- simulateResiduals(modelPrePlantWeeds)
plot(simres)
```

```
#emmeans(modelPrePlantWeeds, pairwise~cover_crop|Year:Field,type="response")
```

ANOVA Table reporting the effects of insecticide treatment and cover
crop on preplant cover

Statistical results for weed biomass in May/June

| Model:  Weeds\_g/0.75 ~ Treatment + cover\_crop \* Field \* Year + (1 | Plot\_ID:Field) + (1 | Block:Field) + (1 | Plot:Field) | | | |
| --- | --- | --- | --- |
| N = 204 | | | |
| Model significance:   Chisq = 83.05 , df = 13 , P = 0 | | | |
| Distribution used: lognormal | | | |
| Conditional Nakagawa R2 (including random effects): 0 | | Marginal Nakagawa R2 (fixed effects only): 0 | |
| Fixed effect term | Chisq | Df | Pr(>Chisq) |
| (Intercept) | 46.519 | 1 | 0.000 |
| Treatment | 2.800 | 2 | 0.247 |
| cover\_crop | 5.982 | 1 | 0.014 |
| Field | 31.152 | 1 | 0.000 |
| Year | 17.466 | 2 | 0.000 |
| cover\_crop:Field | 4.278 | 1 | 0.039 |
| cover\_crop:Year | 2.042 | 2 | 0.360 |
| Field:Year | 35.955 | 2 | 0.000 |
| cover\_crop:Field:Year | 0.961 | 2 | 0.618 |
|  |  |  |  |
| --- | --- | --- | --- |
| Estimate of Random effects   Plot\_ID:Field - var = 0.043   Block:Field - var = 0.067   Plot:Field - var = 0.014 | | | |

Output the pairwise comparisons

```
emmeans(modelPrePlantWeeds, pairwise~cover_crop|Field:Year,type="response")
```

```
## $emmeans
## Field = N, Year = 2017:
##  cover_crop response   SE  df asymp.LCL asymp.UCL
##  cover          6.58 1.78 Inf      3.87      11.2
##  fallow        13.23 3.10 Inf      8.37      20.9
## 
## Field = S, Year = 2017:
##  cover_crop response   SE  df asymp.LCL asymp.UCL
##  cover         36.52 6.61 Inf     25.61      52.1
##  fallow        36.35 7.12 Inf     24.76      53.4
## 
## Field = N, Year = 2018:
##  cover_crop response   SE  df asymp.LCL asymp.UCL
##  cover          8.03 2.26 Inf      4.62      13.9
##  fallow         9.35 2.35 Inf      5.71      15.3
## 
## Field = S, Year = 2018:
##  cover_crop response   SE  df asymp.LCL asymp.UCL
##  cover         36.19 6.57 Inf     25.36      51.7
##  fallow        29.99 5.89 Inf     20.41      44.1
## 
## Field = N, Year = 2019:
##  cover_crop response   SE  df asymp.LCL asymp.UCL
##  cover         17.58 3.74 Inf     11.59      26.7
##  fallow        32.87 6.05 Inf     22.92      47.1
## 
## Field = S, Year = 2019:
##  cover_crop response   SE  df asymp.LCL asymp.UCL
##  cover         17.13 3.67 Inf     11.26      26.1
##  fallow        23.86 4.65 Inf     16.28      35.0
## 
## Results are averaged over the levels of: Treatment 
## Confidence level used: 0.95 
## Intervals are back-transformed from the log scale 
## 
## $contrasts
## Field = N, Year = 2017:
##  contrast       ratio    SE  df null z.ratio p.value
##  cover / fallow 0.497 0.142 Inf    1  -2.446  0.0145
## 
## Field = S, Year = 2017:
##  contrast       ratio    SE  df null z.ratio p.value
##  cover / fallow 1.005 0.182 Inf    1   0.026  0.9795
## 
## Field = N, Year = 2018:
##  contrast       ratio    SE  df null z.ratio p.value
##  cover / fallow 0.859 0.268 Inf    1  -0.487  0.6259
## 
## Field = S, Year = 2018:
##  contrast       ratio    SE  df null z.ratio p.value
##  cover / fallow 1.207 0.234 Inf    1   0.972  0.3312
## 
## Field = N, Year = 2019:
##  contrast       ratio    SE  df null z.ratio p.value
##  cover / fallow 0.535 0.110 Inf    1  -3.038  0.0024
## 
## Field = S, Year = 2019:
##  contrast       ratio    SE  df null z.ratio p.value
##  cover / fallow 0.718 0.155 Inf    1  -1.533  0.1253
## 
## Results are averaged over the levels of: Treatment 
## Tests are performed on the log scale
```

FIGURE 1: Estimated marginal means (95% confidence intervals [CIs])
of weed biomass (g m-2) before planting in each field and year.
Significance of cover crop (CC) treatments included in panels where GLMM
indicated cover crops had a significant effect by year/field slicing.
Means for treatments with cover crops indicated with a solid line, means
without cover crop indicated by a dashed line. Raw data are shown as
open small shapes behind means and CIs. North fields (N) on top panels,
South fields (S) on bottom panels for each year.

```
## Warning: Removed 13 rows containing missing values or values outside the scale range
## (`geom_point()`).
```

### Total biomass in May/June

```
# Start by creating full model to graph data (with IPM treatment)

modelPrePlantWeeds.full <- glmmTMB(Total.biomass_g/0.75~Treatment*cover_crop*Year*Field+(1|Plot_ID:Field)+(1|Block:Field)+(1|Plot:Field),family="lognormal",data=subset(preplant.cover.data)) 
#summary(modelPrePlantWeeds.full)
#Anova(modelPrePlantWeeds.full,type=3)

simres <- simulateResiduals(modelPrePlantWeeds.full) # full model violates assumptions 
plot(simres)
```

```
x=emmeans(modelPrePlantWeeds.full, ~cover_crop:Treatment:Field:Year,type="response")

biomass.preplant.table = as.data.frame(x)
```

Reduce non-significant interactions. The results of this model are
reported in the text. Note: some interactions are not significant but
they help with model fit, and are thus retained.

```
modelPrePlantWeeds <- glmmTMB(Total.biomass_g/0.75~Treatment*Year*Field+cover_crop*Year*Field+(1|Plot_ID:Field)+(1|Block:Field)+(1|Plot:Field),family="lognormal",data=subset(preplant.cover.data)) 
summaryOutput <- summary(modelPrePlantWeeds)
anovaOutput <- Anova(modelPrePlantWeeds,type=3)
r2Output <- r2_nakagawa(modelPrePlantWeeds)

# test the fit of fixed factors 
modelPrePlantWeeds.null <- glmmTMB(Total.biomass_g/0.75~1+(1|Plot_ID:Field)+(1|Block:Field)+(1|Plot:Field),family=lognormal(link='log'),data=subset(preplant.cover.data)) 
model_null_comp <- anova(modelPrePlantWeeds,modelPrePlantWeeds.null)

simres <- simulateResiduals(modelPrePlantWeeds)
plot(simres)
```

```
emmeans(modelPrePlantWeeds, pairwise~cover_crop|Field:Year,type="response")
```

```
## $emmeans
## Field = N, Year = 2017:
##  cover_crop response   SE  df asymp.LCL asymp.UCL
##  cover         30.11 4.06 Inf     23.11      39.2
##  fallow        10.41 2.01 Inf      7.14      15.2
## 
## Field = S, Year = 2017:
##  cover_crop response   SE  df asymp.LCL asymp.UCL
##  cover         46.23 5.52 Inf     36.58      58.4
##  fallow        32.29 4.95 Inf     23.91      43.6
## 
## Field = N, Year = 2018:
##  cover_crop response   SE  df asymp.LCL asymp.UCL
##  cover         28.47 3.82 Inf     21.88      37.0
##  fallow         7.84 1.61 Inf      5.24      11.7
## 
## Field = S, Year = 2018:
##  cover_crop response   SE  df asymp.LCL asymp.UCL
##  cover         51.33 5.94 Inf     40.91      64.4
##  fallow        24.23 3.83 Inf     17.77      33.0
## 
## Field = N, Year = 2019:
##  cover_crop response   SE  df asymp.LCL asymp.UCL
##  cover         46.72 5.28 Inf     37.44      58.3
##  fallow        28.00 3.94 Inf     21.25      36.9
## 
## Field = S, Year = 2019:
##  cover_crop response   SE  df asymp.LCL asymp.UCL
##  cover         33.26 4.35 Inf     25.75      43.0
##  fallow        20.20 3.05 Inf     15.02      27.2
## 
## Results are averaged over the levels of: Treatment 
## Confidence level used: 0.95 
## Intervals are back-transformed from the log scale 
## 
## $contrasts
## Field = N, Year = 2017:
##  contrast       ratio    SE  df null z.ratio p.value
##  cover / fallow  2.89 0.567 Inf    1   5.414  <.0001
## 
## Field = S, Year = 2017:
##  contrast       ratio    SE  df null z.ratio p.value
##  cover / fallow  1.43 0.233 Inf    1   2.209  0.0272
## 
## Field = N, Year = 2018:
##  contrast       ratio    SE  df null z.ratio p.value
##  cover / fallow  3.63 0.747 Inf    1   6.276  <.0001
## 
## Field = S, Year = 2018:
##  contrast       ratio    SE  df null z.ratio p.value
##  cover / fallow  2.12 0.350 Inf    1   4.540  <.0001
## 
## Field = N, Year = 2019:
##  contrast       ratio    SE  df null z.ratio p.value
##  cover / fallow  1.67 0.242 Inf    1   3.528  0.0004
## 
## Field = S, Year = 2019:
##  contrast       ratio    SE  df null z.ratio p.value
##  cover / fallow  1.65 0.271 Inf    1   3.031  0.0024
## 
## Results are averaged over the levels of: Treatment 
## Tests are performed on the log scale
```

ANOVA Table reporting the effects of insecticide treatment and cover
crop on preplant cover

Statistical results for total biomass in May/June

| Model:  Total.biomass\_g/0.75 ~ Treatment \* Year \* Field + cover\_crop \* Year \* Field + (1 | Plot\_ID:Field) + (1 | Block:Field) + (1 | Plot:Field) | | | |
| --- | --- | --- | --- |
| N = 209 | | | |
| Model significance:   Chisq = 159.2 , df = 23 , P = 0 | | | |
| Distribution used: lognormal | | | |
| Conditional Nakagawa R2 (including random effects): 0 | | Marginal Nakagawa R2 (fixed effects only): 0 | |
| Fixed effect term | Chisq | Df | Pr(>Chisq) |
| (Intercept) | 315.238 | 1 | 0.000 |
| Treatment | 0.072 | 2 | 0.965 |
| Year | 4.641 | 2 | 0.098 |
| Field | 3.177 | 1 | 0.075 |
| cover\_crop | 29.316 | 1 | 0.000 |
| Treatment:Year | 3.478 | 4 | 0.481 |
| Treatment:Field | 1.181 | 2 | 0.554 |
| Year:Field | 16.284 | 2 | 0.000 |
| Year:cover\_crop | 11.979 | 2 | 0.003 |
| Field:cover\_crop | 7.733 | 1 | 0.005 |
| Treatment:Year:Field | 3.673 | 4 | 0.452 |
| Year:Field:cover\_crop | 5.406 | 2 | 0.067 |
|  |  |  |  |
| --- | --- | --- | --- |
| Estimate of Random effects   Plot\_ID:Field - var = 0.018   Block:Field - var = 0.03   Plot:Field - var = 0 | | | |

FIGURE S1:Estimated marginal means (95% confidence intervals [CIs])
of total pre-plant biomass (g m-2) before planting in each field and
year. Significance of cover crop treatments (CC) included in panels
where GLM indicated cover crops had a significant effect by year/field
slicing. Means for treatments with cover crops indicated with a solid
line, means without cover crop indicated by a dashed line. Raw data
shown as open small shapes behind means and CIs

```
## Warning: Removed 7 rows containing missing values or values outside the scale range
## (`geom_point()`).
```
