# Supplementary material for: Insecticides may facilitate the escape of weeds from biological control
Source: PeerJ. 2025 Jan 13;13:e18597. doi: 10.7717/peerj.18597 (PMC11737330; doi:10.7717/peerj.18597)
Supplement: Supplemental Information 4 [file peerj-13-18597-s004.html]

Weed Seed Bank Rowen et al. 2024


# Weed Seed Bank Rowen et al. 2024

#### Elizabeth Rowen

#### 5/8/2024

# Context for this document

This document includes the code for Rowen et al. submitted to Field
Crops Research in 2024 titled ” Insecticides may facilitate the escape
of weeds from biological control”

**This includes the code and statistical output for the
Pre-plant cover data. The code covers Results section 3.2, Fig 2&3,
and Fig S2&3.**

**Abstract** 1. CONTEXT: Preventative pesticide seed
treatments (hereafter preventative pest management or PPM) are common in
large acreage row crops such as corn and soybean, and often include
active ingredients of both fungicides and neonicotinoid insecticides.
While PPM is intended to protect crops from soil-borne pathogens and
early season insect pests, these seed treatments may have detrimental
effects on biological control of weed seeds by insects.  
2. RESEARCH QUESTION: Here, in two 3-year corn-soy rotations in
Pennsylvania USA, we investigated a PPM approach to insect management
compared to an integrated pest management approach (IPM) and a “no
(insect) pest management” (NPM) control. This was crossed with a grass
cover crop treatment to see if this conservation practice can help
recover some of the ecosystem services affected by chemical pest
management practices. We hypothesized that PPM and IPM approaches would
release weed seeds from biological control by insects, resulting in
higher weed pressure, but cover crops would increase biological
control.  
3. METHODS: We measured the effect of these treatments on granivorous
insect activity-density, weed-seed predation, the weed-seed bank, and
mid-season weed biomass.  
4. RESULTS: We found that, contrary to our hypothesis, planting a cover
crop decreased carabid activity-density, but did not have consistent
differences in weed-seed predation. Pest management and cover crop
treatments also had inconsistent effects on the weed-seed bank and
mid-season weed biomass, but insecticide use did affect the biomass of
glyphosate-resistant marestail (*Erigeron canadensis* L.). At the
end of the trial, glyphosate-resistant marestail was more abundant in
IPM and PPM plots without a cover crop.  
5. IMPLICATIONS: Our results suggest that reducing insecticide use may
be important when combating herbicide-resistant weeds. As the adoption
conservation agriculture, including the use of no-till and cover crops,
grows, so does the use of neonicotinoid seed treatments and herbicides.
Planting cover crops and/or avoiding the use of insecticides may combat
these problematic weeds.;

### Set-up

Import libraries

```
# importing and formatting data 
library(readxl)
library(plyr)
library(flextable)
library(reshape2)

# creating figures 

library(ggplot2)
library(patchwork)
# Running models 
library(car)
library(glmmTMB)
```

```
## Warning in check_dep_version(dep_pkg = "TMB"): package version mismatch: 
## glmmTMB was built with TMB package version 1.9.15
## Current TMB package version is 1.9.14
## Please re-install glmmTMB from source or restore original 'TMB' package (see '?reinstalling' for more information)
```

```
library(DHARMa)
library(vegan)
library(pscl)
#library(VGAM)
library(performance)
library(corrplot)
library(emmeans)
```

Set up colors for graphs

```
colors = c("seagreen","darkseagreen4","darkgoldenrod4", "darkgoldenrod3","tomato4","tomato3") 
colors.treat = c("seagreen","darkgoldenrod3","tomato3") # only for insecticide treatments 
colors.2017 = c("#085C3D","seagreen","#FC9078","#FFE2DB") # for 2017 when IPM had not yet been implemented 
# c("seagreen","darkseagreen4","tomato3","tomato4")
colors.cc <- c("darkgrey","antiquewhite")
```

Import data

```
wsb.data=read_excel("~/Dropbox/1PhD/Smith & Wickings Field Experiments (AKA smickings)/Manuscripts in progress/Weeds/PeerJ/Data&Stats/2_weedseedbank_2017_2018_2019_Apr2021.xlsx",sheet="weedseedbank_2017_2018_2019_Apr")
wsb.data$TimeYear <- wsb.data$Year
wsb.data$Year <- as.factor(wsb.data$Year)
wsb.data=mutate(wsb.data,Treatment=ifelse(Plot_ID=='105N' | Plot_ID=='106N'| Plot_ID=='203N'| Plot_ID=='204N'|Plot_ID=='305N'| Plot_ID=='306N'| 
                                                        Plot_ID=='401N'|Plot_ID=='402N'| Plot_ID=='501N'| Plot_ID=='504N'| Plot_ID=='602N'| Plot_ID=='603N'| 
                                                        Plot_ID=='102S'| Plot_ID=='106S'| Plot_ID=='201S'| Plot_ID=='204S'| Plot_ID=='303S'| Plot_ID=='305S'| 
                                                        Plot_ID=='402S'| Plot_ID=='406S'| Plot_ID=='501S'| Plot_ID=='504S'| Plot_ID=='603S'| Plot_ID=='605S','1NPM', 
                                                      ifelse(Plot_ID=='101N' | Plot_ID=='104N'| Plot_ID=='202N'| Plot_ID=='205N'| Plot_ID=='301N'| Plot_ID=='303N'| 
                                                               Plot_ID=='405N'| Plot_ID=='406N'| Plot_ID=='502N'| Plot_ID=='503N'| Plot_ID=='604N'| Plot_ID=='606N'| 
                                                               Plot_ID=='104S'| Plot_ID=='105S' | Plot_ID=='202S'| Plot_ID=='203S'| Plot_ID=='301S'| 
                                                               Plot_ID=='306S'| Plot_ID=='404S'| Plot_ID=='405S'| Plot_ID=='502S'| Plot_ID=='503S'| Plot_ID=='601S'| 
                                                               Plot_ID=='606S','2IPM','3PPM')))

wsb.data=mutate(wsb.data,cover_crop=ifelse(Plot_ID=='101N' | Plot_ID=='102N'| Plot_ID=='106N'| Plot_ID=='203N'|Plot_ID=='205N'| Plot_ID=='206N'| Plot_ID=='303N'| Plot_ID=='304N'| 
                                                         Plot_ID=='305N'| Plot_ID=='401N'| Plot_ID=='403N'| Plot_ID=='406N'| Plot_ID=='502N'| Plot_ID=='504N'|Plot_ID=='505N'| Plot_ID=='601N'| 
                                                         Plot_ID=='602N'| Plot_ID=='604N'| Plot_ID=='102S'| Plot_ID=='103S'| Plot_ID=='105S'| Plot_ID=='202S'| Plot_ID=='204S'| Plot_ID=='206S'|
                                                         Plot_ID=='303S'| Plot_ID=='304S'| Plot_ID=='306S'| Plot_ID=='401S'|  Plot_ID=='404S'| Plot_ID=='406S'| Plot_ID=='501S'| Plot_ID=='503S'|
                                                         Plot_ID=='505S'| Plot_ID=='601S'| Plot_ID=='602S'|Plot_ID=='605S','cover', 'fallow'))
wsb.data=mutate(wsb.data,Treated=ifelse(Year == '2017' &Treatment=='2IPM'|Treatment== '1NPM','1NPM',Treatment))
wsb.data$Year=as.factor(wsb.data$Year)
#head(wsb.data)
```

Fig S2 Weed community in the weed seed bank. Stacked bars are colored
by weed species with > 20 individuals recovered over three years.
Grey stripes show one direction of blocking in Latin-square experimental
design. Species abbreviations: Cabo = ˆCapsella bursa-pastoris
(Shepard’s purse), Cah = Cardamine hirsuta (hairy bittercress), Cha =
Chenopodium album (lambsquarter), Coc = Conyza canadensis (marestail),
Osx = Oxalis stricta (yellow woodsorrel) Poo = Portulaca oleracea
(purslane), Sop = Solanum ptychanthum (eastern black nightshade),Ta =
Taraxacum spp. (dandelion), Dii = Digitaria ischaemum (smooth
crabgrass), Pand = Panicum dichotomiflorum (fall panicum), Sef = Setaria
faberi (giant foxtail), Sev = Setaria viridis (green foxtail).
### Weed stats for each field year (Table 1)

```
# Forb biomass 
forb.summary <- ddply(wsb.data, .(Year, Field), summarize, mean.forb.count = mean (forbs), N = sum(!is.na(forbs)),sd=sd(forbs),se=sd/sqrt(N));forb.summary
```

```
##   Year Field mean.forb.count  N        sd        se
## 1 2017     N       60.083333 36 39.795459 6.6325765
## 2 2017     S       57.361111 36 27.446731 4.5744551
## 3 2018     N       39.166667 36 23.462432 3.9104053
## 4 2018     S       31.805556 36 25.156447 4.1927412
## 5 2019     N       17.000000 36 17.019317 2.8365528
## 6 2019     S        2.916667 36  3.228224 0.5380373
```

```
grass.summary <- ddply(wsb.data, .(Year, Field), summarize, mean.grass.count = mean (grasses), N = sum(!is.na(grasses)),sd=sd(grasses),se=sd/sqrt(N));grass.summary
```

```
##   Year Field mean.grass.count  N         sd         se
## 1 2017     N        3.2222222 36  3.0529715 0.50882858
## 2 2017     S       66.5000000 36 51.8594254 8.64323756
## 3 2018     N        1.7222222 36  2.7423776 0.45706293
## 4 2018     S       15.7777778 36 15.3122008 2.55203346
## 5 2019     N        0.1944444 36  0.4671766 0.07786277
## 6 2019     S        3.1944444 36  4.3148875 0.71914791
```

```
richness.summary <-  ddply(wsb.data, .(Year, Field), summarize, mean.richness = mean (total_richness_wsb,na.rm=T), N = sum(!is.na(total_richness_wsb)),sd=sd(total_richness_wsb,na.rm=T),se=sd/sqrt(N));richness.summary
```

```
##   Year Field mean.richness  N       sd        se
## 1 2017     N      6.361111 36 2.598382 0.4330636
## 2 2017     S     10.944444 36 2.848001 0.4746669
## 3 2018     N      6.305556 36 2.670682 0.4451136
## 4 2018     S      7.527778 36 3.307879 0.5513132
## 5 2019     N      3.638889 36 2.058471 0.3430784
## 6 2019     S      2.333333 36 1.820518 0.3034197
```

### Abundance of forbs across treatments

To analyze data, we used a 4-way interaction model with Treatment,
cover crop, year and field and random effects: Plot ID (experimental
unit), and blocking factors Block (column) and Plot (row in Latin-square
design).

```
weed.forbs.overall=glmmTMB(forbs~Treatment*cover_crop*Year*Field+(1|Plot_ID:Field)+(1|Block:Field)+(1|Plot:Field),ziformula=~1, family="genpois", data=subset(wsb.data))
#summary(weed.forbs.overall)
#Anova(weed.forbs.overall,type=3)

simres <- simulateResiduals(weed.forbs.overall)
plot(simres)
```

```
x=emmeans(weed.forbs.overall,~cover_crop:Treatment:Year:Field,type="response")
forb.WSB.table = as.data.frame(x)

#check for multicollinarity between predictors
weed.forbs.overall_noINT <- glmmTMB(forbs~Treatment+cover_crop+Year+Field+(1|Plot_ID:Field)+(1|Block:Field)+(1|Plot:Field),ziformula=~1, family="genpois", data=subset(wsb.data))

check_collinearity(weed.forbs.overall_noINT)
```

```
## # Check for Multicollinearity
## 
## * conditional component:
## 
## Low Correlation
## 
##        Term  VIF       VIF 95% CI Increased SE Tolerance Tolerance 95% CI
##   Treatment 1.01 [1.00, 6.95e+05]         1.00      0.99     [0.00, 1.00]
##  cover_crop 1.01 [1.00,  1461.10]         1.01      0.99     [0.00, 1.00]
##        Year 1.02 [1.00,     6.35]         1.01      0.98     [0.16, 1.00]
##       Field 1.01 [1.00, 6.93e+05]         1.00      0.99     [0.00, 1.00]
```

Reducing non-significant interactions. The results of this model are
reported in the text (section 3.2). Note: some interactions are not
significant but they help with model fit, and are thus retained.

```
weed.forbs.selected=glmmTMB(forbs~Treatment+cover_crop+Year*Field+(1|Plot_ID:Field)+(1|Block:Field)+(1|Plot:Field),ziformula=~1, family="genpois", data=subset(wsb.data))
summaryOutput <- summary(weed.forbs.selected)
anovaOutput <- Anova(weed.forbs.selected,type=3)
r2Output <- r2_nakagawa(weed.forbs.selected)


emmeans(weed.forbs.selected,pairwise~Treatment|Year,type="response")
```

```
## $emmeans
## Year = 2017:
##  Treatment response   SE  df asymp.LCL asymp.UCL
##  1NPM         65.46 7.24 Inf     52.71      81.3
##  2IPM         51.73 6.05 Inf     41.14      65.1
##  3PPM         55.07 6.18 Inf     44.19      68.6
## 
## Year = 2018:
##  Treatment response   SE  df asymp.LCL asymp.UCL
##  1NPM         39.49 4.67 Inf     31.33      49.8
##  2IPM         31.21 3.90 Inf     24.43      39.9
##  3PPM         33.22 4.17 Inf     25.98      42.5
## 
## Year = 2019:
##  Treatment response   SE  df asymp.LCL asymp.UCL
##  1NPM          9.72 1.49 Inf      7.20      13.1
##  2IPM          7.68 1.23 Inf      5.61      10.5
##  3PPM          8.17 1.32 Inf      5.96      11.2
## 
## Results are averaged over the levels of: cover_crop, Field 
## Confidence level used: 0.95 
## Intervals are back-transformed from the log scale 
## 
## $contrasts
## Year = 2017:
##  contrast    ratio     SE  df null z.ratio p.value
##  1NPM / 2IPM 1.265 0.1247 Inf    1   2.390  0.0445
##  1NPM / 3PPM 1.189 0.1137 Inf    1   1.807  0.1673
##  2IPM / 3PPM 0.939 0.0967 Inf    1  -0.607  0.8163
## 
## Year = 2018:
##  contrast    ratio     SE  df null z.ratio p.value
##  1NPM / 2IPM 1.265 0.1247 Inf    1   2.390  0.0445
##  1NPM / 3PPM 1.189 0.1137 Inf    1   1.807  0.1673
##  2IPM / 3PPM 0.939 0.0967 Inf    1  -0.607  0.8163
## 
## Year = 2019:
##  contrast    ratio     SE  df null z.ratio p.value
##  1NPM / 2IPM 1.265 0.1247 Inf    1   2.390  0.0445
##  1NPM / 3PPM 1.189 0.1137 Inf    1   1.807  0.1673
##  2IPM / 3PPM 0.939 0.0967 Inf    1  -0.607  0.8163
## 
## Results are averaged over the levels of: cover_crop, Field 
## P value adjustment: tukey method for comparing a family of 3 estimates 
## Tests are performed on the log scale
```

```
simres <- simulateResiduals(weed.forbs.selected)
plot(simres)
```

```
# test the fit of fixed factors 
weed.forbs.null <- glmmTMB(forbs~1+(1|Plot_ID:Field)+(1|Block:Field)+(1|Plot:Field),ziformula=~1, family="genpois", data=subset(wsb.data))
model_null_comp <- anova(weed.forbs.selected,weed.forbs.null)
```

ANOVA Table reporting the effects of insecticide treatment and cover
crop on forb abundance in weed seed bank

Statistical results for WSB forb abundance including 2017

| Model:  forbs ~ Treatment + cover\_crop + Year \* Field + (1 | Plot\_ID:Field) + (1 | Block:Field) + (1 | Plot:Field) | | | |
| --- | --- | --- | --- |
| N = 216 | | | |
| Model significance:   Chisq = 217.39 , df = 8 , P = 0 | | | |
| Distribution used: genpois | | | |
| Conditional Nakagawa R2 (including random effects): 0.97 | | Marginal Nakagawa R2 (fixed effects only): 0.89 | |
| Fixed effect term | Chisq | Df | Pr(>Chisq) |
| (Intercept) | 690.263 | 1 | 0.000 |
| Treatment | 6.425 | 2 | 0.040 |
| cover\_crop | 1.599 | 1 | 0.206 |
| Year | 61.028 | 2 | 0.000 |
| Field | 0.176 | 1 | 0.675 |
| Year:Field | 28.144 | 2 | 0.000 |
|  |  |  |  |
| --- | --- | --- | --- |
| Estimate of Random effects   Plot\_ID:Field - var = 0   Block:Field - var = 0.034   Plot:Field - var = 0.039 | | | |

```
weed.forbs.selected=glmmTMB(forbs~Treatment+cover_crop+Year*Field+(1|Plot_ID:Field)+(1|Block:Field)+(1|Plot:Field),ziformula=~1, family="genpois", data=subset(wsb.data,Year!="2017"))
summaryOutput <- summary(weed.forbs.selected)
anovaOutput <- Anova(weed.forbs.selected,type=3)
r2Output <- r2_nakagawa(weed.forbs.selected)


emmeans(weed.forbs.selected,pairwise~Treatment|Year,type="response")
```

```
## $emmeans
## Year = 2018:
##  Treatment response   SE  df asymp.LCL asymp.UCL
##  1NPM         35.79 4.58 Inf     27.86      46.0
##  2IPM         30.04 4.19 Inf     22.85      39.5
##  3PPM         37.55 4.74 Inf     29.32      48.1
## 
## Year = 2019:
##  Treatment response   SE  df asymp.LCL asymp.UCL
##  1NPM          8.62 1.41 Inf      6.26      11.9
##  2IPM          7.23 1.28 Inf      5.11      10.2
##  3PPM          9.04 1.53 Inf      6.49      12.6
## 
## Results are averaged over the levels of: cover_crop, Field 
## Confidence level used: 0.95 
## Intervals are back-transformed from the log scale 
## 
## $contrasts
## Year = 2018:
##  contrast    ratio    SE  df null z.ratio p.value
##  1NPM / 2IPM 1.192 0.164 Inf    1   1.275  0.4095
##  1NPM / 3PPM 0.953 0.127 Inf    1  -0.361  0.9308
##  2IPM / 3PPM 0.800 0.114 Inf    1  -1.564  0.2616
## 
## Year = 2019:
##  contrast    ratio    SE  df null z.ratio p.value
##  1NPM / 2IPM 1.192 0.164 Inf    1   1.275  0.4095
##  1NPM / 3PPM 0.953 0.127 Inf    1  -0.361  0.9308
##  2IPM / 3PPM 0.800 0.114 Inf    1  -1.564  0.2616
## 
## Results are averaged over the levels of: cover_crop, Field 
## P value adjustment: tukey method for comparing a family of 3 estimates 
## Tests are performed on the log scale
```

```
simres <- simulateResiduals(weed.forbs.selected)
plot(simres)
```

```
# test the fit of fixed factors 
weed.forbs.null <- glmmTMB(forbs~1+(1|Plot_ID:Field)+(1|Block:Field)+(1|Plot:Field),ziformula=~1, family="genpois", data=subset(wsb.data,Year!="2017"))
model_null_comp <- anova(weed.forbs.selected,weed.forbs.null)
```

ANOVA Table reporting the effects of insecticide treatment and cover
crop on forb abundance in weed seed bank

Statistical results for WSB forb abundance excluding 2017

| Model:  forbs ~ Treatment + cover\_crop + Year \* Field + (1 | Plot\_ID:Field) + (1 | Block:Field) + (1 | Plot:Field) | | | |
| --- | --- | --- | --- |
| Model significance:   Chisq = 114.68 , df = 6 , P = 0 | | | |
| Distribution used: genpois | | | |
| Fixed effect term | Chisq | Df | Pr(>Chisq) |
| (Intercept) | 453.612 | 1 | 0.000 |
| Treatment | 2.690 | 2 | 0.261 |
| cover\_crop | 5.018 | 1 | 0.025 |
| Year | 31.922 | 1 | 0.000 |
| Field | 1.878 | 1 | 0.171 |
| Year:Field | 16.488 | 1 | 0.000 |
|  |  |  |  |
| --- | --- | --- | --- |
| Estimate of Random effects   Plot\_ID:Field - var = 0   Block:Field - var = 0.02   Plot:Field - var = 0.047 | | | |

Figure 2: : A) Estimated marginal means (95% confidence intervals
[CIs]) of forb abundance (per 946 cm3 soil) in the weed-seed bank before
planting in each field and year, and B) for 2018 and 2019 and fields
combined. Means for treatments with cover crops indicated with a solid
line, means without cover crop indicated by a dashed line. Raw data
shown as open small shapes behind means and CIs

### Abundance of grass across treatments

To analyze data, we used a 4-way interaction model with Treatment,
cover crop, year and field and random effects: Plot ID (experimental
unit), and blocking factors Block (column) and Plot (row in Latin-square
design).

```
weed.grasses.overall=glmmTMB(grasses~Treatment*cover_crop*Field*Year+(1|Plot_ID:Field)+(1|Block:Field)+(1|Plot:Field),ziformula=~1, family="genpois", data=wsb.data)
#summary(weed.grasses.overall)
#Anova(weed.grasses.overall,type=3)
simres <- simulateResiduals(weed.grasses.overall)
plot(simres)
```

```
emmeans(weed.grasses.overall,pairwise~cover_crop|Year:Field)
```

```
## NOTE: Results may be misleading due to involvement in interactions
```

```
## $emmeans
## Year = 2017, Field = N:
##  cover_crop emmean       SE  df asymp.LCL asymp.UCL
##  cover       1.239    0.325 Inf  6.03e-01     1.875
##  fallow      1.094    0.338 Inf  4.31e-01     1.757
## 
## Year = 2018, Field = N:
##  cover_crop emmean       SE  df asymp.LCL asymp.UCL
##  cover       0.656    0.379 Inf -8.66e-02     1.398
##  fallow      0.524    0.375 Inf -2.11e-01     1.259
## 
## Year = 2019, Field = N:
##  cover_crop emmean       SE  df asymp.LCL asymp.UCL
##  cover      -0.733    0.574 Inf -1.86e+00     0.393
##  fallow     -6.550 1368.206 Inf -2.69e+03  2675.084
## 
## Year = 2017, Field = S:
##  cover_crop emmean       SE  df asymp.LCL asymp.UCL
##  cover       4.202    0.214 Inf  3.78e+00     4.622
##  fallow      3.842    0.219 Inf  3.41e+00     4.271
## 
## Year = 2018, Field = S:
##  cover_crop emmean       SE  df asymp.LCL asymp.UCL
##  cover       2.486    0.257 Inf  1.98e+00     2.989
##  fallow      2.634    0.246 Inf  2.15e+00     3.116
## 
## Year = 2019, Field = S:
##  cover_crop emmean       SE  df asymp.LCL asymp.UCL
##  cover       0.922    0.349 Inf  2.38e-01     1.607
##  fallow      1.189    0.310 Inf  5.81e-01     1.798
## 
## Results are averaged over the levels of: Treatment 
## Results are given on the log (not the response) scale. 
## Confidence level used: 0.95 
## 
## $contrasts
## Year = 2017, Field = N:
##  contrast       estimate       SE  df z.ratio p.value
##  cover - fallow    0.145    0.356 Inf   0.406  0.6848
## 
## Year = 2018, Field = N:
##  contrast       estimate       SE  df z.ratio p.value
##  cover - fallow    0.132    0.434 Inf   0.304  0.7611
## 
## Year = 2019, Field = N:
##  contrast       estimate       SE  df z.ratio p.value
##  cover - fallow    5.818 1368.206 Inf   0.004  0.9966
## 
## Year = 2017, Field = S:
##  contrast       estimate       SE  df z.ratio p.value
##  cover - fallow    0.360    0.111 Inf   3.238  0.0012
## 
## Year = 2018, Field = S:
##  contrast       estimate       SE  df z.ratio p.value
##  cover - fallow   -0.148    0.206 Inf  -0.718  0.4729
## 
## Year = 2019, Field = S:
##  contrast       estimate       SE  df z.ratio p.value
##  cover - fallow   -0.267    0.349 Inf  -0.764  0.4452
## 
## Results are averaged over the levels of: Treatment 
## Results are given on the log (not the response) scale.
```

```
x=emmeans(weed.grasses.overall,~cover_crop:Treatment:Year:Field,type="response")
grass.WSB.table = as.data.frame(x)
grass.WSB.table
```

```
##  cover_crop Treatment Year Field response        SE  df asymp.LCL asymp.UCL
##  cover      1NPM      2017 N      3.14993  1.543554 Inf   1.20556         8
##  fallow     1NPM      2017 N      2.00258  1.185838 Inf   0.62740         6
##  cover      2IPM      2017 N      2.83838  1.417413 Inf   1.06660         8
##  fallow     2IPM      2017 N      3.00300  1.431021 Inf   1.18014         8
##  cover      3PPM      2017 N      4.59517  1.911397 Inf   2.03347        10
##  fallow     3PPM      2017 N      4.42741  1.858701 Inf   1.94447        10
##  cover      1NPM      2018 N      1.16977  0.826896 Inf   0.29268         5
##  fallow     1NPM      2018 N      1.41942  0.853760 Inf   0.43664         5
##  cover      2IPM      2018 N      2.83219  1.414501 Inf   1.06414         8
##  fallow     2IPM      2018 N      2.12896  1.109134 Inf   0.76685         6
##  cover      3PPM      2018 N      2.15771  1.106250 Inf   0.78993         6
##  fallow     3PPM      2018 N      1.59268  0.927207 Inf   0.50884         5
##  cover      1NPM      2019 N      0.42399  0.431114 Inf   0.05779         3
##  fallow     1NPM      2019 N      0.00000  0.000090 Inf   0.00000       Inf
##  cover      2IPM      2019 N      0.71993  0.538388 Inf   0.16624         3
##  fallow     2IPM      2019 N      0.36549  0.375901 Inf   0.04869         3
##  cover      3PPM      2019 N      0.36363  0.374063 Inf   0.04842         3
##  fallow     3PPM      2019 N      0.36507  0.375666 Inf   0.04858         3
##  cover      1NPM      2017 S     74.08217 17.391094 Inf  46.76174       117
##  fallow     1NPM      2017 S     49.17459 12.126195 Inf  30.32766        80
##  cover      2IPM      2017 S     70.15562 16.530394 Inf  44.20775       111
##  fallow     2IPM      2017 S     52.44448 12.818481 Inf  32.48248        85
##  cover      3PPM      2017 S     57.41624 13.875083 Inf  35.75487        92
##  fallow     3PPM      2017 S     39.31731 10.092417 Inf  23.77320        65
##  cover      1NPM      2018 S     16.98584  5.101000 Inf   9.42896        31
##  fallow     1NPM      2018 S     14.88226  4.743518 Inf   7.96820        28
##  cover      2IPM      2018 S     11.30606  3.762911 Inf   5.88860        22
##  fallow     2IPM      2018 S     13.52035  4.209479 Inf   7.34466        25
##  cover      3PPM      2018 S      9.03211  3.146998 Inf   4.56259        18
##  fallow     3PPM      2018 S     13.43956  4.215667 Inf   7.26748        25
##  cover      1NPM      2019 S      2.42529  1.265234 Inf   0.87238         7
##  fallow     1NPM      2019 S      4.72439  1.899120 Inf   2.14871        10
##  cover      2IPM      2019 S      1.96385  1.128988 Inf   0.63645         6
##  fallow     2IPM      2019 S      2.08919  1.000345 Inf   0.81735         5
##  cover      3PPM      2019 S      3.34060  1.493806 Inf   1.39058         8
##  fallow     3PPM      2019 S      3.58960  1.581141 Inf   1.51394         9
## 
## Confidence level used: 0.95 
## Intervals are back-transformed from the log scale
```

```
weed.grasses.null <- glmmTMB(grasses~1+(1|Plot_ID:Field)+(1|Block:Field)+(1|Plot:Field),ziformula=~1, family="genpois", data=wsb.data)
model_null_comp <- anova(weed.grasses.overall,weed.grasses.null)
model_null_comp
```

```
## Data: wsb.data
## Models:
## weed.grasses.null: grasses ~ 1 + (1 | Plot_ID:Field) + (1 | Block:Field) + (1 | , zi=~1, disp=~1
## weed.grasses.null:     Plot:Field), zi=~1, disp=~1
## weed.grasses.overall: grasses ~ Treatment * cover_crop * Field * Year + (1 | Plot_ID:Field) + , zi=~1, disp=~1
## weed.grasses.overall:     (1 | Block:Field) + (1 | Plot:Field), zi=~1, disp=~1
##                      Df    AIC    BIC  logLik deviance  Chisq Chi Df Pr(>Chisq)
## weed.grasses.null     6 1348.0 1368.3 -668.02   1336.0                         
## weed.grasses.overall 41 1184.7 1323.1 -551.36   1102.7 233.31     35  < 2.2e-16
##                         
## weed.grasses.null       
## weed.grasses.overall ***
## ---
## Signif. codes:  0 '***' 0.001 '**' 0.01 '*' 0.05 '.' 0.1 ' ' 1
```

Reducing non-significant interactions. The results of this model are
reported in the text (section 3.2). Note: some interactions are not
significant but they help with model fit, and are thus retained.

```
weed.grasses.selected=glmmTMB(grasses~Treatment+cover_crop+Field*Year+(1|Plot_ID:Field)+(1|Block:Field)+(1|Plot:Field),ziformula=~1, family="genpois", data=wsb.data)

summaryOutput <- summary(weed.grasses.selected)
anovaOutput <-Anova(weed.grasses.selected,type=3)
r2Output <- r2_nakagawa(weed.grasses.selected)

simres <- simulateResiduals(weed.grasses.selected)
plot(simres)
```

```
#(weed.grasses.selected,pairwise~Treatment|Year,type="response")


# test the fit of fixed factors 
weed.grasses.null <- glmmTMB(grasses~1+(1|Plot_ID:Field)+(1|Block:Field)+(1|Plot:Field),ziformula=~1, family="genpois", data=wsb.data)
model_null_comp <- anova(weed.grasses.selected,weed.grasses.null)
```

ANOVA Table reporting the effects of insecticide treatment and cover
crop on forb abundance in weed seed bank

Statistical results for WSB grasses abundance including 2017

| Model:  grasses ~ Treatment + cover\_crop + Field \* Year + (1 | Plot\_ID:Field) + (1 | Block:Field) + (1 | Plot:Field) | | | |
| --- | --- | --- | --- |
| N = 216 | | | |
| Model significance:   Chisq = 212.82 , df = 8 , P = 0 | | | |
| Distribution used: genpois | | | |
| Conditional Nakagawa R2 (including random effects): 0.97 | | Marginal Nakagawa R2 (fixed effects only): 0.9 | |
| Fixed effect term | Chisq | Df | Pr(>Chisq) |
| (Intercept) | 28.284 | 1 | 0.000 |
| Treatment | 2.157 | 2 | 0.340 |
| cover\_crop | 5.304 | 1 | 0.021 |
| Field | 72.290 | 1 | 0.000 |
| Year | 23.769 | 2 | 0.000 |
| Field:Year | 9.362 | 2 | 0.009 |
|  |  |  |  |
| --- | --- | --- | --- |
| Estimate of Random effects   Plot\_ID:Field - var = 0   Block:Field - var = 0.138   Plot:Field - var = 0.07 | | | |

Remove 2017

```
weed.grasses.selectedno2017=glmmTMB(grasses~Treatment+cover_crop+Year+Field+(1|Plot_ID:Field)+(1|Block:Field)+(1|Plot:Field),ziformula=~1, family="genpois", data=subset(wsb.data,Year!="2017"))
summaryOutput <- summary(weed.grasses.selectedno2017)
anovaOutput <- Anova(weed.grasses.selectedno2017,type=3)
r2Output <- r2_nakagawa(weed.grasses.selectedno2017)


#emmeans(weed.forbs.selected,pairwise~Treatment|Year,type="response")

simres <- simulateResiduals(weed.grasses.selectedno2017)
plot(simres)
```

```
# test the fit of fixed factors 
weed.grasses.nullno2017 <- glmmTMB(grasses~1+(1|Plot_ID:Field)+(1|Block:Field)+(1|Plot:Field),ziformula=~1, family="genpois", data=subset(wsb.data,Year!="2017"))
model_null_comp <- anova(weed.grasses.selectedno2017,weed.grasses.nullno2017)
```

ANOVA Table reporting the effects of insecticide treatment and cover
crop on forb abundance in weed seed bank

Statistical results for WSB grasses abundance excluding 2017

| Model:  grasses ~ Treatment + cover\_crop + Year + Field + (1 | Plot\_ID:Field) + (1 | Block:Field) + (1 | Plot:Field) | | | |
| --- | --- | --- | --- |
| N = 144 | | | |
| Model significance:   Chisq = 86.29 , df = 5 , P = 0 | | | |
| Distribution used: genpois | | | |
| Conditional Nakagawa R2 (including random effects): 0.92 | | Marginal Nakagawa R2 (fixed effects only): 0.82 | |
| Fixed effect term | Chisq | Df | Pr(>Chisq) |
| (Intercept) | 3.510 | 1 | 0.061 |
| Treatment | 1.231 | 2 | 0.540 |
| cover\_crop | 0.086 | 1 | 0.770 |
| Year | 69.086 | 1 | 0.000 |
| Field | 51.292 | 1 | 0.000 |
|  |  |  |  |
| --- | --- | --- | --- |
| Estimate of Random effects   Plot\_ID:Field - var = 0.103   Block:Field - var = 0.102   Plot:Field - var = 0.01 | | | |

Figure 3: Estimated marginal means (95% confidence intervals [CIs])
of grass abundance (per 946 cm3 soil) in the weed-seed bank before
planting A) for in each field and year, and B) for all years and fields
combined. In A) means for treatments with cover crops indicated with a
solid line, means without cover crop indicated by a dashed line. Raw
data shown as open small shapes behind means and CIs

### Species richness weed seed bank before planting

```
wsb.data$total_richness_wsb<- specnumber(wsb.data[8:60])
#summary(wsb.data)

weed.richness.overall=glmmTMB(total_richness_wsb~Year*Field*Treatment*cover_crop+(1|Plot_ID:Field)+(1|Block:Field)+(1|Plot:Field)+(1|Plot_ID:Field),family='genpois', data=wsb.data)
#summary(weed.richness.overall)
#Anova(weed.richness.overall,type=3)

simres <- simulateResiduals(weed.richness.overall)
plot(simres)
```

```
x=emmeans(weed.richness.overall,~cover_crop:Treatment:Year:Field,type="response")
richness.WSB.table = as.data.frame(x)
```

```
weed.richness.selected=glmmTMB(total_richness_wsb~Year*Field+Treatment+cover_crop+(1|Plot_ID:Field)+(1|Block:Field)+(1|Plot:Field),family='genpois',  data=wsb.data)

summaryOutput <- summary(weed.richness.selected)
anovaOutput <-Anova(weed.richness.selected,type=3)
#r2Output <- r2_nakagawa(weed.richness.selected)

simres <- simulateResiduals(weed.richness.selected)
plot(simres)
```

```
emmeans(weed.richness.selected,~cover_crop:Treatment|Year:Field,type="response")
```

```
## Year = 2017, Field = N:
##  cover_crop Treatment response    SE  df asymp.LCL asymp.UCL
##  cover      1NPM          6.54 0.677 Inf      5.34      8.01
##  fallow     1NPM          6.91 0.713 Inf      5.64      8.45
##  cover      2IPM          5.42 0.570 Inf      4.41      6.66
##  fallow     2IPM          5.72 0.601 Inf      4.66      7.03
##  cover      3PPM          6.46 0.668 Inf      5.28      7.92
##  fallow     3PPM          6.82 0.704 Inf      5.57      8.35
## 
## Year = 2018, Field = N:
##  cover_crop Treatment response    SE  df asymp.LCL asymp.UCL
##  cover      1NPM          6.49 0.673 Inf      5.30      7.96
##  fallow     1NPM          6.85 0.707 Inf      5.60      8.39
##  cover      2IPM          5.38 0.567 Inf      4.38      6.61
##  fallow     2IPM          5.68 0.596 Inf      4.62      6.97
##  cover      3PPM          6.41 0.665 Inf      5.23      7.86
##  fallow     3PPM          6.77 0.699 Inf      5.53      8.29
## 
## Year = 2019, Field = N:
##  cover_crop Treatment response    SE  df asymp.LCL asymp.UCL
##  cover      1NPM          3.75 0.435 Inf      2.99      4.71
##  fallow     1NPM          3.96 0.458 Inf      3.15      4.96
##  cover      2IPM          3.11 0.366 Inf      2.47      3.91
##  fallow     2IPM          3.28 0.386 Inf      2.60      4.13
##  cover      3PPM          3.70 0.430 Inf      2.95      4.65
##  fallow     3PPM          3.91 0.452 Inf      3.11      4.90
## 
## Year = 2017, Field = S:
##  cover_crop Treatment response    SE  df asymp.LCL asymp.UCL
##  cover      1NPM         11.07 1.061 Inf      9.17     13.35
##  fallow     1NPM         11.68 1.118 Inf      9.68     14.09
##  cover      2IPM          9.17 0.895 Inf      7.57     11.10
##  fallow     2IPM          9.67 0.944 Inf      7.99     11.71
##  cover      3PPM         10.93 1.050 Inf      9.05     13.19
##  fallow     3PPM         11.53 1.106 Inf      9.56     13.92
## 
## Year = 2018, Field = S:
##  cover_crop Treatment response    SE  df asymp.LCL asymp.UCL
##  cover      1NPM          7.64 0.769 Inf      6.27      9.30
##  fallow     1NPM          8.06 0.810 Inf      6.62      9.81
##  cover      2IPM          6.33 0.649 Inf      5.17      7.74
##  fallow     2IPM          6.68 0.684 Inf      5.46      8.16
##  cover      3PPM          7.54 0.761 Inf      6.19      9.19
##  fallow     3PPM          7.96 0.801 Inf      6.53      9.69
## 
## Year = 2019, Field = S:
##  cover_crop Treatment response    SE  df asymp.LCL asymp.UCL
##  cover      1NPM          2.39 0.312 Inf      1.85      3.09
##  fallow     1NPM          2.52 0.328 Inf      1.95      3.25
##  cover      2IPM          1.98 0.261 Inf      1.53      2.56
##  fallow     2IPM          2.09 0.275 Inf      1.61      2.70
##  cover      3PPM          2.36 0.309 Inf      1.83      3.05
##  fallow     3PPM          2.49 0.324 Inf      1.93      3.21
## 
## Confidence level used: 0.95 
## Intervals are back-transformed from the log scale
```

ANOVA Table reporting the effects of insecticide treatment and cover
crop on forb abundance in weed seed bank

Statistical results for WSB richness including 2017

| Model:  total\_richness\_wsb ~ Year \* Field + Treatment + cover\_crop + (1 | Plot\_ID:Field) + (1 | Block:Field) + (1 | Plot:Field) | | | |
| --- | --- | --- | --- |
| N = 216 | | | |
| Model significance:   Chisq = 86.29 , df = 5 , P = 0 | | | |
| Distribution used: genpois | | | |
| Fixed effect term | Chisq | Df | Pr(>Chisq) |
| (Intercept) | 330.084 | 1 | 0.000 |
| Year | 36.616 | 2 | 0.000 |
| Field | 17.016 | 1 | 0.000 |
| Treatment | 10.781 | 2 | 0.005 |
| cover\_crop | 1.117 | 1 | 0.291 |
| Year:Field | 43.502 | 2 | 0.000 |
|  |  |  |  |
| --- | --- | --- | --- |
| Estimate of Random effects   Plot\_ID:Field - var = 0   Block:Field - var = 0.022   Plot:Field - var = 0.009 | | | |

Figure S3: Estimated marginal means (95% confidence intervals [CIs])
of species richness in the weed-seed bank before planting A) in each
field and year, and B) for all years and fields combined. In A) means
for treatments with cover crops indicated with a solid line, means
without cover crop indicated by a dashed line. Raw data shown as open
small shapes behind means and CIs. Significance of PM treatments
included in panels where GLMM indicated PM had a significant effect, and
the P values for pairwise comparisons are given.
