# Supplementary material for: Insecticides may facilitate the escape of weeds from biological control
Source: PeerJ. 2025 Jan 13;13:e18597. doi: 10.7717/peerj.18597 (PMC11737330; doi:10.7717/peerj.18597)
Supplement: Supplemental Information 6 [file peerj-13-18597-s006.html]

Weed Biomass August Rowen 2024


# Weed Biomass August Rowen 2024

#### Elizabeth Rowen

#### 5/8/2024

# Context for this document

This document includes the code for Rowen et al. submitted to Field
Crops Research in 2024 titled ” Insecticides may facilitate the escape
of weeds from biological control”

**This includes the code and statistical output for the
Pre-plant cover data. The code covers Results section 3.3, Fig 4&5,
and Fig S4&5.**

**Abstract** 1. CONTEXT: Preventative pesticide seed
treatments (hereafter preventative pest management or PPM) are common in
large acreage row crops such as corn and soybean, and often include
active ingredients of both fungicides and neonicotinoid insecticides.
While PPM is intended to protect crops from soil-borne pathogens and
early season insect pests, these seed treatments may have detrimental
effects on biological control of weed seeds by insects.  
2. RESEARCH QUESTION: Here, in two 3-year corn-soy rotations in
Pennsylvania USA, we investigated a PPM approach to insect management
compared to an integrated pest management approach (IPM) and a “no
(insect) pest management” (NPM) control. This was crossed with a grass
cover crop treatment to see if this conservation practice can help
recover some of the ecosystem services affected by chemical pest
management practices. We hypothesized that PPM and IPM approaches would
release weed seeds from biological control by insects, resulting in
higher weed pressure, but cover crops would increase biological
control.  
3. METHODS: We measured the effect of these treatments on granivorous
insect activity-density, weed-seed predation, the weed-seed bank, and
mid-season weed biomass.  
4. RESULTS: We found that, contrary to our hypothesis, planting a cover
crop decreased carabid activity-density, but did not have consistent
differences in weed-seed predation. Pest management and cover crop
treatments also had inconsistent effects on the weed-seed bank and
mid-season weed biomass, but insecticide use did affect the biomass of
glyphosate-resistant marestail (*Erigeron canadensis* L.). At the
end of the trial, glyphosate-resistant marestail was more abundant in
IPM and PPM plots without a cover crop.  
5. IMPLICATIONS: Our results suggest that reducing insecticide use may
be important when combating herbicide-resistant weeds. As the adoption
conservation agriculture, including the use of no-till and cover crops,
grows, so does the use of neonicotinoid seed treatments and herbicides.
Planting cover crops and/or avoiding the use of insecticides may combat
these problematic weeds.;

### Set-up

Import libraries

```
# importing and formatting data 
library(readxl)
library(plyr)
library(flextable)
library(reshape2)
library(tidyr)

# creating figures 

library(ggplot2)
library(patchwork)
# Running models 
library(car)
library(glmmTMB)
library(DHARMa)
library(vegan)
library(pscl)
#library(VGAM)
library(performance)
library(corrplot)
library(emmeans)
```

Set up colors for graphs

```
colors = c("seagreen","darkseagreen4","darkgoldenrod4", "darkgoldenrod3","tomato4","tomato3") 
colors.treat = c("seagreen","darkgoldenrod3","tomato3") # only for insecticide treatments 
colors.2017 = c("#085C3D","seagreen","#FC9078","#FFE2DB") # for 2017 when IPM had not yet been implemented 
# c("seagreen","darkseagreen4","tomato3","tomato4")
colors.cc <- c("darkgrey","antiquewhite")
```

```
# import data 
Aug.weeds.data=read_excel('~/Dropbox/1PhD/Smith & Wickings Field Experiments (AKA smickings)/Manuscripts in progress/Weeds/PeerJ/Revision/To upload/Data&Stats/3_AugWeed biomass_Apr2021.xlsx',sheet='Sheet1')
Aug.weeds.data$TimeYear <- Aug.weeds.data$Year
Aug.weeds.data$Year <- as.factor(Aug.weeds.data$Year )

# add treatment details  
Aug.weeds.data=mutate(Aug.weeds.data,Treatment=ifelse(Plot_ID=='105N' | Plot_ID=='106N'| Plot_ID=='203N'| Plot_ID=='204N'|Plot_ID=='305N'| Plot_ID=='306N'| 
                                                        Plot_ID=='401N'|Plot_ID=='402N'| Plot_ID=='501N'| Plot_ID=='504N'| Plot_ID=='602N'| Plot_ID=='603N'| 
                                                        Plot_ID=='102S'| Plot_ID=='106S'| Plot_ID=='201S'| Plot_ID=='204S'| Plot_ID=='303S'| Plot_ID=='305S'| 
                                                        Plot_ID=='402S'| Plot_ID=='406S'| Plot_ID=='501S'| Plot_ID=='504S'| Plot_ID=='603S'| Plot_ID=='605S','1NPM', 
                                                      ifelse(Plot_ID=='101N' | Plot_ID=='104N'| Plot_ID=='202N'| Plot_ID=='205N'| Plot_ID=='301N'| Plot_ID=='303N'| 
                                                               Plot_ID=='405N'| Plot_ID=='406N'| Plot_ID=='502N'| Plot_ID=='503N'| Plot_ID=='604N'| Plot_ID=='606N'| 
                                                               Plot_ID=='104S'| Plot_ID=='105S' | Plot_ID=='202S'| Plot_ID=='203S'| Plot_ID=='301S'| 
                                                               Plot_ID=='306S'| Plot_ID=='404S'| Plot_ID=='405S'| Plot_ID=='502S'| Plot_ID=='503S'| Plot_ID=='601S'| 
                                                               Plot_ID=='606S','2IPM','3PPM')))

Aug.weeds.data=mutate(Aug.weeds.data,cover_crop=ifelse(Plot_ID=='101N' | Plot_ID=='102N'| Plot_ID=='106N'| Plot_ID=='203N'|Plot_ID=='205N'| Plot_ID=='206N'| Plot_ID=='303N'| Plot_ID=='304N'| 
                                                         Plot_ID=='305N'| Plot_ID=='401N'| Plot_ID=='403N'| Plot_ID=='406N'| Plot_ID=='502N'| Plot_ID=='504N'|Plot_ID=='505N'| Plot_ID=='601N'| 
                                                         Plot_ID=='602N'| Plot_ID=='604N'| Plot_ID=='102S'| Plot_ID=='103S'| Plot_ID=='105S'| Plot_ID=='202S'| Plot_ID=='204S'| Plot_ID=='206S'|
                                                         Plot_ID=='303S'| Plot_ID=='304S'| Plot_ID=='306S'| Plot_ID=='401S'|  Plot_ID=='404S'| Plot_ID=='406S'| Plot_ID=='501S'| Plot_ID=='503S'|
                                                         Plot_ID=='505S'| Plot_ID=='601S'| Plot_ID=='602S'|Plot_ID=='605S','cover', 'fallow'))
Aug.weeds.data=mutate(Aug.weeds.data,Treated=ifelse(Year == '2017' &Treatment=='2IPM'|Treatment== '1NPM','1NPM',Treatment))
Aug.weeds.data$total_weeds_aug=Aug.weeds.data$Total.Biomass

Aug.weeds.data$richness=rowSums(Aug.weeds.data[,6:53]>0)
Aug.weeds.data$forb.richness=rowSums(Aug.weeds.data[,6:45]>0)
Aug.weeds.data$yllw.Wood.sorrel <- as.numeric(Aug.weeds.data$`yllw Wood-sorrel`)
Aug.weeds.data$Redroot.Pigwd<- as.numeric(Aug.weeds.data$`Redroot Pigwd`)
```

### Weed stats for each field year (Table 1)

```
# Forb biomass 
forb.summary <- ddply(Aug.weeds.data, .(Year, Field), summarize, mean.forb.biomass = mean (forbs), N = sum(!is.na(forbs)),sd=sd(forbs),se=sd/sqrt(N));forb.summary
```

```
##   Year Field mean.forb.biomass  N        sd        se
## 1 2017     N          2.638333 36  2.969503 0.4949171
## 2 2017     S         10.283333 36 13.331927 2.2219879
## 3 2018     N          8.183333 36 11.932493 1.9887488
## 4 2018     S         18.727500 36 21.322312 3.5537187
## 5 2019     N          6.840556 36  9.052256 1.5087093
## 6 2019     S          7.728056 36 13.754658 2.2924429
```

```
# grass biomass 
grass.summary <- ddply(Aug.weeds.data, .(Year, Field), summarize, mean.grass.biomass = mean (grass), N = sum(!is.na(grass)),sd=sd(grass),se=sd/sqrt(N));grass.summary
```

```
##   Year Field mean.grass.biomass  N         sd         se
## 1 2017     N          1.5675000 36  4.0561286 0.67602143
## 2 2017     S         37.6416667 36 26.9456954 4.49094923
## 3 2018     N          0.1388889 36  0.3323318 0.05538864
## 4 2018     S         92.5694444 36 53.4216907 8.90361512
## 5 2019     N         86.5316667 36 43.9268142 7.32113571
## 6 2019     S          4.0869444 36  6.1537713 1.02562856
```

```
# Forb richness
forb.richness.summary <-  ddply(Aug.weeds.data, .(Year, Field), summarize, mean.forb.richness = mean (forb.richness,na.rm=T), N = sum(!is.na(forb.richness)),sd=sd(forb.richness,na.rm=T),se=sd/sqrt(N));forb.richness.summary
```

```
##   Year Field mean.forb.richness  N       sd        se
## 1 2017     N           3.111111 36 1.115547 0.1859245
## 2 2017     S           2.138889 36 1.641476 0.2735794
## 3 2018     N           3.241379 29 1.353703 0.2513764
## 4 2018     S           6.000000 25 1.581139 0.3162278
## 5 2019     N           5.694444 36 1.653040 0.2755066
## 6 2019     S           2.805556 36 1.064208 0.1773681
```

### Weed community across the plots in each year (Fig S4)

### Biomass of forbs across treatments

```
## full model for graphing 

weed.forbs.all=glmmTMB(forbs/0.75~Treatment*cover_crop*Year*Field+(1|Plot_ID:Field)+(1|Block:Field)+(1|Plot:Field),zi=~1,family=ziGamma(link="log"), data=Aug.weeds.data)
summary(weed.forbs.all)
```

```
##  Family: Gamma  ( log )
## Formula:          
## forbs/0.75 ~ Treatment * cover_crop * Year * Field + (1 | Plot_ID:Field) +  
##     (1 | Block:Field) + (1 | Plot:Field)
## Zero inflation:              ~1
## Data: Aug.weeds.data
## 
##      AIC      BIC   logLik deviance df.resid 
##   1513.3   1651.7   -715.7   1431.3      175 
## 
## Random effects:
## 
## Conditional model:
##  Groups        Name        Variance  Std.Dev.
##  Plot_ID:Field (Intercept) 7.925e-05 0.008902
##  Block:Field   (Intercept) 9.324e-02 0.305356
##  Plot:Field    (Intercept) 3.769e-02 0.194147
## Number of obs: 216, groups:  Plot_ID:Field, 72; Block:Field, 12; Plot:Field, 12
## 
## Dispersion estimate for Gamma family (sigma^2): 1.08 
## 
## Conditional model:
##                                                Estimate Std. Error z value
## (Intercept)                                      1.0912     0.4549   2.399
## Treatment2IPM                                   -0.1573     0.6068  -0.259
## Treatment3PPM                                   -0.4141     0.6149  -0.673
## cover_cropfallow                                 0.6884     0.6172   1.115
## Year2018                                         1.7467     0.6383   2.736
## Year2019                                         1.4513     0.6344   2.288
## FieldS                                           1.8379     0.6787   2.708
## Treatment2IPM:cover_cropfallow                  -0.6009     0.8687  -0.692
## Treatment3PPM:cover_cropfallow                  -0.3507     0.8751  -0.401
## Treatment2IPM:Year2018                          -0.8987     0.8826  -1.018
## Treatment3PPM:Year2018                          -0.1766     0.9078  -0.195
## Treatment2IPM:Year2019                          -0.4916     0.9208  -0.534
## Treatment3PPM:Year2019                          -1.7003     0.9496  -1.790
## cover_cropfallow:Year2018                       -1.4887     0.8804  -1.691
## cover_cropfallow:Year2019                       -0.5135     0.8776  -0.585
## Treatment2IPM:FieldS                            -0.4097     0.9278  -0.442
## Treatment3PPM:FieldS                            -0.5273     0.9093  -0.580
## cover_cropfallow:FieldS                         -0.4482     0.9241  -0.485
## Year2018:FieldS                                 -1.7886     0.9068  -1.972
## Year2019:FieldS                                 -2.7512     0.9237  -2.979
## Treatment2IPM:cover_cropfallow:Year2018          2.6753     1.2650   2.115
## Treatment3PPM:cover_cropfallow:Year2018          0.7147     1.2398   0.577
## Treatment2IPM:cover_cropfallow:Year2019          1.3646     1.3889   0.982
## Treatment3PPM:cover_cropfallow:Year2019          2.5844     1.3135   1.968
## Treatment2IPM:cover_cropfallow:FieldS            0.8254     1.3203   0.625
## Treatment3PPM:cover_cropfallow:FieldS            0.5815     1.2928   0.450
## Treatment2IPM:Year2018:FieldS                    1.3514     1.2832   1.053
## Treatment3PPM:Year2018:FieldS                    2.1132     1.2957   1.631
## Treatment2IPM:Year2019:FieldS                    1.8919     1.2958   1.460
## Treatment3PPM:Year2019:FieldS                    3.3130     1.3051   2.538
## cover_cropfallow:Year2018:FieldS                 1.9098     1.2692   1.505
## cover_cropfallow:Year2019:FieldS                 0.7531     1.2948   0.582
## Treatment2IPM:cover_cropfallow:Year2018:FieldS  -3.0806     1.8215  -1.691
## Treatment3PPM:cover_cropfallow:Year2018:FieldS  -3.0967     1.7836  -1.736
## Treatment2IPM:cover_cropfallow:Year2019:FieldS  -2.3337     1.9374  -1.204
## Treatment3PPM:cover_cropfallow:Year2019:FieldS  -2.9289     1.8280  -1.602
##                                                Pr(>|z|)   
## (Intercept)                                     0.01646 * 
## Treatment2IPM                                   0.79548   
## Treatment3PPM                                   0.50070   
## cover_cropfallow                                0.26466   
## Year2018                                        0.00621 **
## Year2019                                        0.02216 * 
## FieldS                                          0.00677 **
## Treatment2IPM:cover_cropfallow                  0.48908   
## Treatment3PPM:cover_cropfallow                  0.68863   
## Treatment2IPM:Year2018                          0.30858   
## Treatment3PPM:Year2018                          0.84574   
## Treatment2IPM:Year2019                          0.59341   
## Treatment3PPM:Year2019                          0.07338 . 
## cover_cropfallow:Year2018                       0.09085 . 
## cover_cropfallow:Year2019                       0.55842   
## Treatment2IPM:FieldS                            0.65883   
## Treatment3PPM:FieldS                            0.56198   
## cover_cropfallow:FieldS                         0.62764   
## Year2018:FieldS                                 0.04856 * 
## Year2019:FieldS                                 0.00290 **
## Treatment2IPM:cover_cropfallow:Year2018         0.03445 * 
## Treatment3PPM:cover_cropfallow:Year2018         0.56431   
## Treatment2IPM:cover_cropfallow:Year2019         0.32588   
## Treatment3PPM:cover_cropfallow:Year2019         0.04912 * 
## Treatment2IPM:cover_cropfallow:FieldS           0.53188   
## Treatment3PPM:cover_cropfallow:FieldS           0.65286   
## Treatment2IPM:Year2018:FieldS                   0.29229   
## Treatment3PPM:Year2018:FieldS                   0.10291   
## Treatment2IPM:Year2019:FieldS                   0.14429   
## Treatment3PPM:Year2019:FieldS                   0.01113 * 
## cover_cropfallow:Year2018:FieldS                0.13242   
## cover_cropfallow:Year2019:FieldS                0.56080   
## Treatment2IPM:cover_cropfallow:Year2018:FieldS  0.09080 . 
## Treatment3PPM:cover_cropfallow:Year2018:FieldS  0.08253 . 
## Treatment2IPM:cover_cropfallow:Year2019:FieldS  0.22838   
## Treatment3PPM:cover_cropfallow:Year2019:FieldS  0.10910   
## ---
## Signif. codes:  0 '***' 0.001 '**' 0.01 '*' 0.05 '.' 0.1 ' ' 1
## 
## Zero-inflation model:
##             Estimate Std. Error z value Pr(>|z|)    
## (Intercept)  -2.3979     0.2462   -9.74   <2e-16 ***
## ---
## Signif. codes:  0 '***' 0.001 '**' 0.01 '*' 0.05 '.' 0.1 ' ' 1
```

```
Anova(weed.forbs.all,type=3)
```

```
## Analysis of Deviance Table (Type III Wald chisquare tests)
## 
## Response: forbs/0.75
##                                  Chisq Df Pr(>Chisq)   
## (Intercept)                     5.7535  1   0.016456 * 
## Treatment                       0.4597  2   0.794647   
## cover_crop                      1.2442  1   0.264659   
## Year                            8.6950  2   0.012939 * 
## Field                           7.3326  1   0.006772 **
## Treatment:cover_crop            0.4815  2   0.786043   
## Treatment:Year                  5.9307  4   0.204385   
## cover_crop:Year                 2.9373  2   0.230233   
## Treatment:Field                 0.3689  2   0.831565   
## cover_crop:Field                0.2353  1   0.627640   
## Year:Field                      9.1444  2   0.010335 * 
## Treatment:cover_crop:Year       9.0047  4   0.060982 . 
## Treatment:cover_crop:Field      0.4162  2   0.812115   
## Treatment:Year:Field            6.6889  4   0.153269   
## cover_crop:Year:Field           2.3190  2   0.313636   
## Treatment:cover_crop:Year:Field 4.6506  4   0.325063   
## ---
## Signif. codes:  0 '***' 0.001 '**' 0.01 '*' 0.05 '.' 0.1 ' ' 1
```

```
simres <- simulateResiduals(weed.forbs.all)
plot(simres)
```

```
x=emmeans(weed.forbs.all, ~cover_crop:Treatment:Year:Field,type="response")

weed.forbs.table = as.data.frame(x)
```

```
weed.forbs.selected=glmmTMB(forbs/0.75~Treatment+cover_crop+Field*Year+(1|Plot_ID:Field)+(1|Block:Field)+(1|Plot:Field),zi=~1,family=ziGamma(link="log"),data=subset(Aug.weeds.data,forbs!="NA"))

summaryOutput <- summary(weed.forbs.selected)
anovaOutput <- Anova(weed.forbs.selected,type=3)
r2Output <- r2_nakagawa(weed.forbs.selected)
model_N <- summaryOutput$nobs

simres <- simulateResiduals(weed.forbs.selected)
plot(simres)
```

```
# test the fit of fixed factors 
weed.forbs.null <- glmmTMB(forbs/0.75~1+(1|Plot_ID:Field)+(1|Block:Field)+(1|Plot:Field),ziformula=~1, family=ziGamma(link="log"),data=subset(Aug.weeds.data,forbs!="NA"))
model_null_comp <- anova(weed.forbs.selected,weed.forbs.null)
```

ANOVA Table reporting the effects of insecticide treatment and cover
crop on forb biomass in August

Statistical results for August forb Biomass

| Model:  forbs/0.75 ~ Treatment + cover\_crop + Field \* Year + (1 | Plot\_ID:Field) + (1 | Block:Field) + (1 | Plot:Field) | | | |
| --- | --- | --- | --- |
| N = 216 | | | |
| Model significance:   Chisq = 43.12 , df = 8 , P = 0 | | | |
| Distribution used: Gamma | | | |
| Conditional Nakagawa R2 (including random effects): 0.4 | | Marginal Nakagawa R2 (fixed effects only): 0.31 | |
| Fixed effect term | Chisq | Df | Pr(>Chisq) |
| (Intercept) | 19.788 | 1 | 0.000 |
| Treatment | 1.289 | 2 | 0.525 |
| cover\_crop | 1.882 | 1 | 0.170 |
| Field | 23.758 | 1 | 0.000 |
| Year | 24.067 | 2 | 0.000 |
| Field:Year | 15.825 | 2 | 0.000 |
|  |  |  |  |
| --- | --- | --- | --- |
| Estimate of Random effects   Plot\_ID:Field - var = 0.07   Block:Field - var = 0.035   Plot:Field - var = 0.014 | | | |

FIG S5: : Estimated marginal means (95% confidence intervals [CIs])
of forb biomass in August for each field and year. Means for treatments
with cover crops indicated with a solid line, means without cover crop
indicated by a dashed line. Raw data shown as open small shapes behind
means and CIs.

### Biomass of Marestail across treatments

```
weed.marestail.2019N=glmmTMB(marestail/0.75~Treatment*cover_crop+(1|Plot_ID)+(1|Block)+(1|Plot),family="nbinom2", data=subset(Aug.weeds.data,Year=="2019"&Field=="N"))

#emmeans(weed.marestail.2019N,pairwise~Treatment:cover_crop,type="response")
#emmeans(weed.marestail.2019N,pairwise~1,type="response")

summaryOutput.2019N <- summary(weed.marestail.2019N)
anovaOutput.2019N <- Anova(weed.marestail.2019N,type=3)
r2Output.2019N <- r2_nakagawa(weed.marestail.2019N)

simres <- simulateResiduals(weed.marestail.2019N)
plot(simres)
```

```
weed.marestail.2019N.null=glmmTMB(marestail/0.75~1+(1|Plot_ID)+(1|Block)+(1|Plot),family="nbinom2", data=subset(Aug.weeds.data,Year=="2019"&Field=="N"))

model_null_comp.2019N <- anova(weed.marestail.2019N,weed.marestail.2019N.null)

weed.marestail.2018N=glmmTMB(marestail/0.75~Treatment+cover_crop+(1|Plot_ID)+(1|Block)+(1|Plot),family="nbinom2",data=subset(Aug.weeds.data,Year=="2018"&Field=="N"))
simres <- simulateResiduals(weed.marestail.2018N)
plot(simres)
```

```
summaryOutput.2018N <- summary(weed.marestail.2018N)
anovaOutput.2018N <- Anova(weed.marestail.2018N,type=3)
r2Output.2018N <- r2_nakagawa(weed.marestail.2018N)

weed.marestail.2018N.null=glmmTMB(marestail/0.75~1+(1|Plot_ID)+(1|Block)+(1|Plot),family="nbinom2", data=subset(Aug.weeds.data,Year=="2018"&Field=="N"))

model_null_comp.2018N <- anova(weed.marestail.2018N,weed.marestail.2018N.null)

weed.marestail.2018S=glmmTMB(marestail/0.75~Treatment+cover_crop+(1|Plot_ID)+(1|Block)+(1|Plot),family="nbinom2",data=subset(Aug.weeds.data,Year=="2018"&Field=="S"))

summaryOutput.2018S <- summary(weed.marestail.2018S)
anovaOutput.2018S <- Anova(weed.marestail.2018S,type=3)
r2Output.2018S <- r2_nakagawa(weed.marestail.2018S)


simres <- simulateResiduals(weed.marestail.2018S)
plot(simres)
```

```
weed.marestail.2018S.null=glmmTMB(marestail/0.75~1+(1|Plot_ID)+(1|Block)+(1|Plot),family="nbinom2", data=subset(Aug.weeds.data,Year=="2018"&Field=="S"))

model_null_comp.2018S <- anova(weed.marestail.2018S,weed.marestail.2018S.null)
```

ANOVA Table reporting the effects of insecticide treatment and cover
crop on Marestail biomass North Field 2019

Statistical results for Marestail biomass North Field 2019

| Model:  marestail/0.75 ~ Treatment \* cover\_crop + (1 | Plot\_ID) + (1 | Block) + (1 | Plot) | | | |
| --- | --- | --- | --- |
| N = 36 | | | |
| Model significance:   Chisq = 13.97 , df = 5 , P = 0.0158 | | | |
| Distribution used: nbinom2 | | | |
| Fixed effect term | Chisq | Df | Pr(>Chisq) |
| (Intercept) | 271.907 | 1 | 0.000 |
| Treatment | 0.118 | 2 | 0.943 |
| cover\_crop | 0.673 | 1 | 0.412 |
| Treatment:cover\_crop | 6.911 | 2 | 0.032 |
|  |  |  |  |
| --- | --- | --- | --- |
| Estimate of Random effects   Plot\_ID - var = 0   Block - var = 0.194   Plot - var = 0.053 | | | |

ANOVA Table reporting the effects of insecticide treatment and cover
crop on Marestail biomass North Field 2018

Statistical results for Marestail biomass North Field 2018

| Model:  marestail/0.75 ~ Treatment + cover\_crop + (1 | Plot\_ID) + (1 | Block) + (1 | Plot) | | | |
| --- | --- | --- | --- |
| N = 35 | | | |
| Model significance:   Chisq = 1.44 , df = 3 , P = 0.696 | | | |
| Distribution used: nbinom2 | | | |
| Fixed effect term | Chisq | Df | Pr(>Chisq) |
| (Intercept) | 27.210 | 1 | 0.000 |
| Treatment | 1.411 | 2 | 0.494 |
| cover\_crop | 0.003 | 1 | 0.958 |
|  |  |  |  |
| --- | --- | --- | --- |
| Estimate of Random effects   Plot\_ID - var = 0.512   Block - var = 0   Plot - var = 0.041 | | | |

ANOVA Table reporting the effects of insecticide treatment and cover
crop on Marestail biomass South Field 2018

Statistical results for Marestail biomass South Field 2018

| Model:  marestail/0.75 ~ Treatment + cover\_crop + (1 | Plot\_ID) + (1 | Block) + (1 | Plot) | | | |
| --- | --- | --- | --- |
| N = 34 | | | |
| Model significance:   Chisq = NA , df = 3 , P = NA | | | |
| Distribution used: nbinom2 | | | |
| Fixed effect term | Chisq | Df | Pr(>Chisq) |
| (Intercept) | 0.976 | 1 | 0.323 |
| Treatment | 0.159 | 2 | 0.923 |
| cover\_crop | 0.012 | 1 | 0.912 |
|  |  |  |  |
| --- | --- | --- | --- |
| Estimate of Random effects   Plot\_ID - var = 34.325   Block - var = 0   Plot - var = 0 | | | |

Figure 4: Estimated marginal means (95% confidence intervals [CIs])
of marestail biomass (g m-2) in August for in each field and year. Means
for treatments with cover crops indicated with a solid line, means
without cover crop indicated by a dashed line. Raw data shown as open
small shapes behind means and CIs.
Spatial autocorrelation between of marestail within plots

```
library(gstat)
library(sp)
library(ape)
```

```
## 
## Attaching package: 'ape'
```

```
## The following object is masked from 'package:flextable':
## 
##     rotate
```

```
ggplot(data=subset(Aug.weeds.data,Year=="2019"&Field=="N"),aes(x=Block*33.5,y=Plot*12.2,fill=marestail/0.75))+geom_tile()
```

```
marestail_autocorrelation_data <- data.frame(x=subset(Aug.weeds.data,Year=="2019"&Field=="N")$Plot*12.2,y=subset(Aug.weeds.data,Year=="2019"&Field=="N")$Block*33.5, marestail = subset(Aug.weeds.data,Year=="2019"&Field=="N")$marestail,trans_marestail = sqrt(subset(Aug.weeds.data,Year=="2019"&Field=="N")$marestail))

hist(marestail_autocorrelation_data$trans_marestail)
```

```
shapiro.test(marestail_autocorrelation_data$trans_marestail)
```

```
## 
##  Shapiro-Wilk normality test
## 
## data:  marestail_autocorrelation_data$trans_marestail
## W = 0.98409, p-value = 0.8724
```

```
shapiro.test(marestail_autocorrelation_data$marestail)
```

```
## 
##  Shapiro-Wilk normality test
## 
## data:  marestail_autocorrelation_data$marestail
## W = 0.96363, p-value = 0.2775
```

```
marestail_autocorrelation_data1 <- marestail_autocorrelation_data

coordinates(marestail_autocorrelation_data1) <-  ~x+y
class(marestail_autocorrelation_data1)
```

```
## [1] "SpatialPointsDataFrame"
## attr(,"package")
## [1] "sp"
```

```
bubble(marestail_autocorrelation_data1, zcol='marestail', fill=TRUE, do.sqrt=FALSE, maxsize=3)
```

```
# raw data 
Vario_RDT=variogram(marestail~1,data=marestail_autocorrelation_data1)  ## Here, we assume that there is a constant trend in the data.
plot(Vario_RDT)
```

```
marestail_var_model <- vgm(psill=1600, model="Sph",range=35, nugget=500)
plot(Vario_RDT,model=marestail_var_model)
```

```
# Moran's I

plot.dists <- as.matrix(dist(cbind(marestail_autocorrelation_data$x, marestail_autocorrelation_data$y)))
plot.dists.inv <- 1/plot.dists 
diag(plot.dists.inv) <- 0
summary(dist(cbind(marestail_autocorrelation_data$x, marestail_autocorrelation_data$y)))
```

```
##    Min. 1st Qu.  Median    Mean 3rd Qu.    Max. 
##   12.20   41.44   68.10   76.46  103.42  178.26
```

```
Moran.I(marestail_autocorrelation_data$marestail, plot.dists.inv)
```

```
## $observed
## [1] 0.01589164
## 
## $expected
## [1] -0.02857143
## 
## $sd
## [1] 0.03308847
## 
## $p.value
## [1] 0.179025
```

```
coordinates <- cbind(marestail_autocorrelation_data$x, marestail_autocorrelation_data$y)
```

### Biomass of grasses across treatments

```
weed.grass.all=glmmTMB(grass/0.75~Treatment*cover_crop+Year*Field+(1|Plot_ID:Field)+(1|Block:Field)+(1|Plot:Field),zi=~1,family=ziGamma(link="log"), data=subset(Aug.weeds.data))
#summary(weed.grass.all)
#Anova(weed.grass.all,type=3)

simres <- simulateResiduals(weed.grass.all)
plot(simres)
```

```
x=emmeans(weed.grass.all, ~cover_crop:Treatment:Field:Year,type="response")

weed.grass.table = as.data.frame(x)
```

Running each Field year separately because North 2018 had very little
grass, and causes errors in an overall model

```
# 2017

## North
weed.grass.2017.N=glmmTMB(round(grass/0.75,0)~Treatment+cover_crop+(1|Block:Field)+(1|Plot:Field),zi=~1,family=nbinom2, data=subset(Aug.weeds.data,Year=="2017"& Field=="N"))
summaryOutput_2017N <- summary(weed.grass.2017.N)
anovaOutput_2017N <- Anova(weed.grass.2017.N,type=3)
r2Output_2017N <- r2_nakagawa(weed.grass.2017.N)

simres <- simulateResiduals(weed.grass.2017.N)
plot(simres)
```

```
weed.grass.2017N.null=glmmTMB(round(grass/0.75,0)~1+(1|Block:Field)+(1|Plot:Field),family="nbinom2", data=subset(Aug.weeds.data,Year=="2017"&Field=="N"))

model_null_comp.2017N <- anova(weed.grass.2017.N,weed.grass.2017N.null)

## South
weed.grass.2017.S=glmmTMB(round(grass/0.75,0)~Treatment+cover_crop+(1|Block:Field)+(1|Plot:Field),zi=~1,family=nbinom2, data=subset(Aug.weeds.data,Year=="2017"& Field=="S"))

summaryOutput_2017S <- summary(weed.grass.2017.S)
anovaOutput_2017S <- Anova(weed.grass.2017.S,type=3)
r2Output_2017S <- r2_nakagawa(weed.grass.2017.S)

emmeans(weed.grass.2017.S,pairwise~Treatment)
```

```
## $emmeans
##  Treatment emmean    SE  df asymp.LCL asymp.UCL
##  1NPM        3.49 0.206 Inf      3.09      3.90
##  2IPM        3.96 0.204 Inf      3.56      4.36
##  3PPM        3.73 0.205 Inf      3.32      4.13
## 
## Results are averaged over the levels of: cover_crop 
## Results are given on the log (not the response) scale. 
## Confidence level used: 0.95 
## 
## $contrasts
##  contrast    estimate    SE  df z.ratio p.value
##  1NPM - 2IPM   -0.461 0.147 Inf  -3.144  0.0047
##  1NPM - 3PPM   -0.231 0.148 Inf  -1.558  0.2642
##  2IPM - 3PPM    0.230 0.145 Inf   1.585  0.2522
## 
## Results are averaged over the levels of: cover_crop 
## Results are given on the log (not the response) scale. 
## P value adjustment: tukey method for comparing a family of 3 estimates
```

```
simres <- simulateResiduals(weed.grass.2017.S)
plot(simres)
```

```
weed.grass.2017S.null=glmmTMB(round(grass/0.75,0)~1+(1|Block:Field)+(1|Plot:Field),family="nbinom2", data=subset(Aug.weeds.data,Year=="2017"&Field=="S"))

model_null_comp.2017S <- anova(weed.grass.2017.S,weed.grass.2017S.null)


# 2018 

## South

weed.grass.2018.S=glmmTMB(round(grass/0.75,0)~Treatment*cover_crop+(1|Block:Field)+(1|Plot:Field),zi=~1,family=nbinom2, data=subset(Aug.weeds.data,Year=="2018"& Field=="S"))
summaryOutput_2018S <- summary(weed.grass.2018.S)
anovaOutput_2018S <- Anova(weed.grass.2018.S,type=3)
r2Output_2018S <- r2_nakagawa(weed.grass.2018.S)

simres <- simulateResiduals(weed.grass.2018.S)
plot(simres)
```

```
weed.grass.2018S.null=glmmTMB(round(grass/0.75,0)~1+(1|Block:Field)+(1|Plot:Field),family="nbinom2", data=subset(Aug.weeds.data,Year=="2018"&Field=="S"))

model_null_comp.2018S <- anova(weed.grass.2018.S,weed.grass.2018S.null)


# 2019
# north 
weed.grass.2019.N=glmmTMB(round(grass/0.75,0)~Treatment+cover_crop+(1|Block:Field)+(1|Plot:Field),zi=~1,family=nbinom2, data=subset(Aug.weeds.data,Year=="2019"& Field=="N"))
summaryOutput_2019N <- summary(weed.grass.2019.N)
anovaOutput_2019N <-Anova(weed.grass.2019.N,type=3)
r2Output_2019N <- r2_nakagawa(weed.grass.2019.N)

simres <- simulateResiduals(weed.grass.2019.N)
plot(simres)
```

```
weed.grass.2019N.null=glmmTMB(round(grass/0.75,0)~1+(1|Block:Field)+(1|Plot:Field),family="nbinom2", data=subset(Aug.weeds.data,Year=="2019"&Field=="N"))

model_null_comp.2019N <- anova(weed.grass.2019.N,weed.grass.2019N.null)


# south 
weed.grass.2019.S=glmmTMB(round(grass/0.75,0)~Treatment+cover_crop+(1|Block:Field)+(1|Plot:Field),zi=~1,family=nbinom2, data=subset(Aug.weeds.data,Year=="2019"& Field=="S"))
summaryOutput_2019S <-summary(weed.grass.2019.S)
anovaOutput_2019S <-Anova(weed.grass.2019.S,type=3)
r2Output_2019S <- r2_nakagawa(weed.grass.2019.S)

simres <- simulateResiduals(weed.grass.2019.S)
plot(simres)
```

```
weed.grass.2019S.null=glmmTMB(round(grass/0.75,0)~1+(1|Block:Field)+(1|Plot:Field),family="nbinom2", data=subset(Aug.weeds.data,Year=="2019"&Field=="S"))

model_null_comp.2019S <- anova(weed.grass.2019.S,weed.grass.2019S.null)
```

ANOVA Table reporting the effects of insecticide treatment and cover
crop on grass biomass North Field 2017

Statistical results for grass biomass North Field 2017

| Model:  round(grass/0.75, 0) ~ Treatment + cover\_crop + (1 | Block:Field) + (1 | Plot:Field) | | | |
| --- | --- | --- | --- |
| N = 36 | | | |
| Model significance:   Chisq = 6.32 , df = 4 , P = 0.1762 | | | |
| Distribution used: nbinom2 | | | |
| Fixed effect term | Chisq | Df | Pr(>Chisq) |
| (Intercept) | 0.068 | 1 | 0.794 |
| Treatment | 2.108 | 2 | 0.349 |
| cover\_crop | 4.803 | 1 | 0.028 |
|  |  |  |  |
| --- | --- | --- | --- |
| Estimate of Random effects   Plot\_ID - var =   Block - var = 1.018   Plot - var = 0 | | | |

ANOVA Table reporting the effects of insecticide treatment and cover
crop on grass biomass South Field 2017

Statistical results for grass biomass South Field 2017

| Model:  round(grass/0.75, 0) ~ Treatment + cover\_crop + (1 | Block:Field) + (1 | Plot:Field) | | | |
| --- | --- | --- | --- |
| N = 36 | | | |
| Model significance:   Chisq = 30.27 , df = 4 , P = 0 | | | |
| Distribution used: nbinom2 | | | |
| Conditional Nakagawa R2 (including random effects): 0.93 | | Marginal Nakagawa R2 (fixed effects only): 0.5 | |
| Fixed effect term | Chisq | Df | Pr(>Chisq) |
| (Intercept) | 201.796 | 1 | 0.000 |
| Treatment | 9.892 | 2 | 0.007 |
| cover\_crop | 48.899 | 1 | 0.000 |
|  |  |  |  |
| --- | --- | --- | --- |
| Estimate of Random effects   Plot\_ID - var =   Block - var = 0.16   Plot - var = 0.028 | | | |

ANOVA Table reporting the effects of insecticide treatment and cover
crop on grass biomass North Field 2018

Statistical results for grass biomass North Field 2018

| Model:  round(grass/0.75, 0) ~ Treatment \* cover\_crop + (1 | Block:Field) + (1 | Plot:Field) | | | |
| --- | --- | --- | --- |
| N = 36 | | | |
| Model significance:   Chisq = 8.07 , df = 6 , P = 0.2334 | | | |
| Distribution used: nbinom2 | | | |
| Conditional Nakagawa R2 (including random effects): 0.96 | | Marginal Nakagawa R2 (fixed effects only): 0.33 | |
| Fixed effect term | Chisq | Df | Pr(>Chisq) |
| (Intercept) | 373.357 | 1 | 0.000 |
| Treatment | 3.019 | 2 | 0.221 |
| cover\_crop | 8.668 | 1 | 0.003 |
| Treatment:cover\_crop | 6.687 | 2 | 0.035 |
|  |  |  |  |
| --- | --- | --- | --- |
| Estimate of Random effects   Plot\_ID - var =   Block - var = 0.097   Plot - var = 0.06 | | | |

ANOVA Table reporting the effects of insecticide treatment and cover
crop on grass biomass North Field 2019

Statistical results for grass biomass North Field 2019

| Model:  round(grass/0.75, 0) ~ Treatment + cover\_crop + (1 | Block:Field) + (1 | Plot:Field) | | | |
| --- | --- | --- | --- |
| N = 36 | | | |
| Model significance:   Chisq = 5.08 , df = 4 , P = 0.2789 | | | |
| Distribution used: nbinom2 | | | |
| Conditional Nakagawa R2 (including random effects): 0.94 | | Marginal Nakagawa R2 (fixed effects only): 0.14 | |
| Fixed effect term | Chisq | Df | Pr(>Chisq) |
| (Intercept) | 447.491 | 1 | 0.000 |
| Treatment | 1.766 | 2 | 0.414 |
| cover\_crop | 3.462 | 1 | 0.063 |
|  |  |  |  |
| --- | --- | --- | --- |
| Estimate of Random effects   Plot\_ID - var =   Block - var = 0.106   Plot - var = 0.044 | | | |

ANOVA Table reporting the effects of insecticide treatment and cover
crop on grass biomass South Field 2019

Statistical results for grass biomass South Field 2017

| Model:  round(grass/0.75, 0) ~ Treatment + cover\_crop + (1 | Block:Field) + (1 | Plot:Field) | | | |
| --- | --- | --- | --- |
| N = 36 | | | |
| Model significance:   Chisq = 12.7 , df = 4 , P = 0.0128 | | | |
| Distribution used: nbinom2 | | | |
| Fixed effect term | Chisq | Df | Pr(>Chisq) |
| (Intercept) | 7.384 | 1 | 0.007 |
| Treatment | 0.779 | 2 | 0.677 |
| cover\_crop | 3.353 | 1 | 0.067 |
|  |  |  |  |
| --- | --- | --- | --- |
| Estimate of Random effects   Plot\_ID - var =   Block - var = 2.257   Plot - var = 0 | | | |

Figure 5: Boxplots of grass biomass (g) in August for in each field
and year. Analyses for each field year were calculated separately.
Significance of treatments included in panels where GLM indicated cover
crops (CC) or pest management (PM) had a significant effect on grass
biomass.
