# Supplementary material for: Insecticides may facilitate the escape of weeds from biological control
Source: PeerJ. 2025 Jan 13;13:e18597. doi: 10.7717/peerj.18597 (PMC11737330; doi:10.7717/peerj.18597)
Supplement: Supplemental Information 8 — Code and statistical output for Results 4, Figures 6, S6 and S7. [file peerj-13-18597-s008.html]

Weed seed predators Rowen 2024


# Weed seed predators Rowen 2024

#### Elizabeth Rowen

#### 5/10/2024

# Context for this document

This document includes the code for Rowen et al. submitted to Field
Crops Research in 2024 titled ” Insecticides may facilitate the escape
of weeds from biological control”

**This includes the code and statistical output for the weed
seed predator data. The code covers Results section 3.4, Fig 6, and Fig
S6&7.**

**Abstract** 1. CONTEXT: Preventative pesticide seed
treatments (hereafter preventative pest management or PPM) are common in
large acreage row crops such as corn and soybean, and often include
active ingredients of both fungicides and neonicotinoid insecticides.
While PPM is intended to protect crops from soil-borne pathogens and
early season insect pests, these seed treatments may have detrimental
effects on biological control of weed seeds by insects.  
2. RESEARCH QUESTION: Here, in two 3-year corn-soy rotations in
Pennsylvania USA, we investigated a PPM approach to insect management
compared to an integrated pest management approach (IPM) and a “no
(insect) pest management” (NPM) control. This was crossed with a grass
cover crop treatment to see if this conservation practice can help
recover some of the ecosystem services affected by chemical pest
management practices. We hypothesized that PPM and IPM approaches would
release weed seeds from biological control by insects, resulting in
higher weed pressure, but cover crops would increase biological
control.  
3. METHODS: We measured the effect of these treatments on granivorous
insect activity-density, weed-seed predation, the weed-seed bank, and
mid-season weed biomass.  
4. RESULTS: We found that, contrary to our hypothesis, planting a cover
crop decreased carabid activity-density, but did not have consistent
differences in weed-seed predation. Pest management and cover crop
treatments also had inconsistent effects on the weed-seed bank and
mid-season weed biomass, but insecticide use did affect the biomass of
glyphosate-resistant marestail (*Erigeron canadensis* L.). At the
end of the trial, glyphosate-resistant marestail was more abundant in
IPM and PPM plots without a cover crop.  
5. IMPLICATIONS: Our results suggest that reducing insecticide use may
be important when combating herbicide-resistant weeds. As the adoption
conservation agriculture, including the use of no-till and cover crops,
grows, so does the use of neonicotinoid seed treatments and herbicides.
Planting cover crops and/or avoiding the use of insecticides may combat
these problematic weeds.;

### Set-up

Import libraries

```
# importing and formatting data 
library(readxl)
library(plyr)
library(flextable)
library(reshape2)
library(tidyr)

# creating figures 

library(ggplot2)
library(patchwork)
# Running models 
library(car)
library(glmmTMB)
library(DHARMa)
library(vegan)
library(pscl)
#library(VGAM)
library(performance)
library(corrplot)
library(emmeans)
```

Set up colors for graphs

```
colors = c("seagreen","darkseagreen4","darkgoldenrod4", "darkgoldenrod3","tomato4","tomato3") 
colors.treat = c("seagreen","darkgoldenrod3","tomato3") # only for insecticide treatments 
colors.2017 = c("#085C3D","seagreen","#FC9078","#FFE2DB") # for 2017 when IPM had not yet been implemented 
# c("seagreen","darkseagreen4","tomato3","tomato4")
colors.cc <- c("darkgrey","antiquewhite")
```

Import data

```
pitfall.fall.data = read_excel('~/Dropbox/1PhD/Smith & Wickings Field Experiments (AKA smickings)/Manuscripts in progress/Weeds/PeerJ/Revision/To upload/Data&Stats/4_Pitfall_data_combined_post-checkOCT2019_Apr2021.xlsx',sheet='Sheet1')
pitfall.fall.data$Year=as.factor(pitfall.fall.data$Year)

# format treatment,cover crop for each plot ID
pitfall.fall.data=mutate(pitfall.fall.data,Treatment=ifelse(Plot_ID=='105N' | Plot_ID=='106N'| Plot_ID=='203N'| Plot_ID=='204N'|Plot_ID=='305N'| Plot_ID=='306N'| 
                                                              Plot_ID=='401N'|Plot_ID=='402N'| Plot_ID=='501N'| Plot_ID=='504N'| Plot_ID=='602N'| Plot_ID=='603N'| 
                                                              Plot_ID=='102S'| Plot_ID=='106S'| Plot_ID=='201S'| Plot_ID=='204S'| Plot_ID=='303S'| Plot_ID=='305S'| 
                                                              Plot_ID=='402S'| Plot_ID=='406S'| Plot_ID=='501S'| Plot_ID=='504S'| Plot_ID=='603S'| Plot_ID=='605S','1NPM', 
                                                            ifelse(Plot_ID=='101N' | Plot_ID=='104N'| Plot_ID=='202N'| Plot_ID=='205N'| Plot_ID=='301N'| Plot_ID=='303N'| 
                                                                     Plot_ID=='405N'| Plot_ID=='406N'| Plot_ID=='502N'| Plot_ID=='503N'| Plot_ID=='604N'| Plot_ID=='606N'| 
                                                                     Plot_ID=='104S'| Plot_ID=='105S' | Plot_ID=='202S'| Plot_ID=='203S'| Plot_ID=='301S'| 
                                                                     Plot_ID=='306S'| Plot_ID=='404S'| Plot_ID=='405S'| Plot_ID=='502S'| Plot_ID=='503S'| Plot_ID=='601S'| 
                                                                     Plot_ID=='606S','2IPM','3PPM')))

pitfall.fall.data=mutate(pitfall.fall.data,cover_crop=ifelse(Plot_ID=='101N' | Plot_ID=='102N'| Plot_ID=='106N'| Plot_ID=='203N'|Plot_ID=='205N'| Plot_ID=='206N'| Plot_ID=='303N'| 
                                                               Plot_ID=='304N'| Plot_ID=='305N'| Plot_ID=='401N'| Plot_ID=='403N'| Plot_ID=='406N'| Plot_ID=='502N'| Plot_ID=='504N'|
                                                               Plot_ID=='505N'| Plot_ID=='601N'| Plot_ID=='602N'| Plot_ID=='604N'| Plot_ID=='102S'| Plot_ID=='103S'| Plot_ID=='105S'| 
                                                               Plot_ID=='202S'| Plot_ID=='204S'| Plot_ID=='206S'| Plot_ID=='303S'| Plot_ID=='304S'| Plot_ID=='306S'| Plot_ID=='401S'| 
                                                               Plot_ID=='404S'| Plot_ID=='406S'| Plot_ID=='501S'| Plot_ID=='503S'| Plot_ID=='505S'| Plot_ID=='601S'| Plot_ID=='602S'|
                                                               Plot_ID=='605S','cover', 'fallow'))

pitfall.fall.data=mutate(pitfall.fall.data,Treated=ifelse(Year == '2017' &Treatment=='2IPM'|Treatment== '1NPM','1NPM',Treatment))


weed.seed.pred.data1 = read_excel('~/Dropbox/1PhD/Smith & Wickings Field Experiments (AKA smickings)/Manuscripts in progress/Weeds/PeerJ/Revision/To upload/Data&Stats/6_WeedSeedPredators.xlsx',sheet='Sheet1')
# format treatment,cover crop for each plot ID
weed.seed.pred.data1=mutate(weed.seed.pred.data1,Treatment=ifelse(Plot_ID=='105N' | Plot_ID=='106N'| Plot_ID=='203N'| Plot_ID=='204N'|Plot_ID=='305N'| Plot_ID=='306N'| 
                                                                    Plot_ID=='401N'|Plot_ID=='402N'| Plot_ID=='501N'| Plot_ID=='504N'| Plot_ID=='602N'| Plot_ID=='603N'| 
                                                                    Plot_ID=='102S'| Plot_ID=='106S'| Plot_ID=='201S'| Plot_ID=='204S'| Plot_ID=='303S'| Plot_ID=='305S'| 
                                                                    Plot_ID=='402S'| Plot_ID=='406S'| Plot_ID=='501S'| Plot_ID=='504S'| Plot_ID=='603S'| Plot_ID=='605S','1NPM', 
                                                                  ifelse(Plot_ID=='101N' | Plot_ID=='104N'| Plot_ID=='202N'| Plot_ID=='205N'| Plot_ID=='301N'| Plot_ID=='303N'| 
                                                                           Plot_ID=='405N'| Plot_ID=='406N'| Plot_ID=='502N'| Plot_ID=='503N'| Plot_ID=='604N'| Plot_ID=='606N'| 
                                                                           Plot_ID=='104S'| Plot_ID=='105S' | Plot_ID=='202S'| Plot_ID=='203S'| Plot_ID=='301S'| 
                                                                           Plot_ID=='306S'| Plot_ID=='404S'| Plot_ID=='405S'| Plot_ID=='502S'| Plot_ID=='503S'| Plot_ID=='601S'| 
                                                                           Plot_ID=='606S','2IPM','3PPM')))
weed.seed.pred.data1=mutate(weed.seed.pred.data1,cover_crop=ifelse(Plot_ID=='101N' | Plot_ID=='102N'| Plot_ID=='106N'| Plot_ID=='203N'|Plot_ID=='205N'| Plot_ID=='206N'| Plot_ID=='303N'| Plot_ID=='304N'| 
                                                                     Plot_ID=='305N'| Plot_ID=='401N'| Plot_ID=='403N'| Plot_ID=='406N'| Plot_ID=='502N'| Plot_ID=='504N'|Plot_ID=='505N'| Plot_ID=='601N'| 
                                                                     Plot_ID=='602N'| Plot_ID=='604N'| Plot_ID=='102S'| Plot_ID=='103S'| Plot_ID=='105S'| Plot_ID=='202S'| Plot_ID=='204S'| Plot_ID=='206S'|
                                                                     Plot_ID=='303S'| Plot_ID=='304S'| Plot_ID=='306S'| Plot_ID=='401S'|  Plot_ID=='404S'| Plot_ID=='406S'| Plot_ID=='501S'| Plot_ID=='503S'|
                                                                     Plot_ID=='505S'| Plot_ID=='601S'| Plot_ID=='602S'|Plot_ID=='605S','cover', 'fallow'))
weed.seed.pred.data1=mutate(weed.seed.pred.data1,Treated=ifelse(Year == '2017' &Treatment=='2IPM'|Treatment== '1NPM','1NPM',Treatment))
weed.seed.pred.data1$Year=as.factor(weed.seed.pred.data1$Year)
weed.seed.pred.data1$predatoryCarabids=pitfall.fall.data$carabids-weed.seed.pred.data1$total_weedPred_carabids
weed.seed.pred.data1$Total_carabids=pitfall.fall.data$carabids
weed.seed.pred.data = ddply(weed.seed.pred.data1,.(Year,Month,Seasonality,Date,Field,Crop,Block,Plot,Plot_ID,Treatment,Treated,cover_crop),summarize,
                            total_weedPred_carabids=mean(total_weedPred_carabids,rm.na=T)*2, 
                            total_bembidion = mean(total_bembidion,rm.na=T)*2,
                            per_bembidion = mean(per_bembidion,rm.na=T),
                            total_harpalus = mean(total_harpalus,rm.na=T)*2,
                           per_harpalus = mean(per_harpalus,rm.na=T),
                           total_amara = mean(total_amara,rm.na=T)*2,
                           per_amara = mean(per_amara,rm.na=T),
                            total_anisodactylus = mean(total_anisodactylus,rm.na=T)*2,
                           per_anisodactylus= mean(per_anisodactylus,rm.na=T),
                           total_notiobia = mean(`Notiobia sayi`,rm.na=T)*2,
                           per_notiobia= mean(per_Notiobia,rm.na=T),
                            total_weedPred=mean(total_weedPred,rm.na=T)*2,
                            ants=mean(ants,rm.na=T)*2,
                           predatoryCarabids=mean(predatoryCarabids,rm.na=T)*2,
                           Total_carabids=mean(Total_carabids,rm.na=T)*2)
```

### Predator stats for each field year (Table 2)

```
carabids.summary <- ddply(weed.seed.pred.data, .(Year, Field,Seasonality), summarize, mean.beetle.count = mean (total_weedPred_carabids,na.rm=T), N = sum(!is.na(total_weedPred_carabids)),sd=sd(total_weedPred_carabids,na.rm=T),se=sd/sqrt(N));carabids.summary
```

```
##    Year Field Seasonality mean.beetle.count  N         sd        se
## 1  2017     N       Early         1.2500000 36  1.6453397 0.2742233
## 2  2017     N        Late         5.9166667 36  8.0759785 1.3459964
## 3  2017     S       Early         1.0555556 36  1.8352804 0.3058801
## 4  2017     S        Late        16.5000000 30 16.1538935 2.9492840
## 5  2018     N       Early         0.4444444 36  0.8086830 0.1347805
## 6  2018     N        Late         2.3333333 36  2.6832816 0.4472136
## 7  2018     S       Early         2.9444444 36  2.0694298 0.3449050
## 8  2018     S        Late        14.4285714 35 15.5303985 2.6251165
## 9  2019     N       Early         0.5277778 36  0.9098229 0.1516372
## 10 2019     N        Late         2.2285714 35  2.0012601 0.3382747
## 11 2019     S       Early         0.6111111 36  1.1533251 0.1922208
## 12 2019     S        Late         0.1428571  7  0.3779645 0.1428571
```

```
ants.summary <- ddply(weed.seed.pred.data, .(Year, Field,Seasonality), summarize, mean.ants.count = mean (ants,na.rm=T), N = sum(!is.na(ants)),sd=sd(ants,na.rm=T),se=sd/sqrt(N));ants.summary
```

```
##    Year Field Seasonality mean.ants.count  N        sd        se
## 1  2017     N       Early       21.444444 36 11.269287 1.8782145
## 2  2017     N        Late        5.944444 36  5.126650 0.8544417
## 3  2017     S       Early        9.638889 36  6.782974 1.1304956
## 4  2017     S        Late        2.700000 30  3.455630 0.6309089
## 5  2018     N       Early       19.416667 36 19.696809 3.2828015
## 6  2018     N        Late        3.666667 36  3.107594 0.5179324
## 7  2018     S       Early       16.222222 36  9.304718 1.5507863
## 8  2018     S        Late        5.600000 35 10.296201 1.7403757
## 9  2019     N       Early        6.888889 36  5.599887 0.9333144
## 10 2019     N        Late        4.742857 35  6.630335 1.1207312
## 11 2019     S       Early       23.888889 36 22.122746 3.6871243
## 12 2019     S        Late       14.714286  7  5.186980 1.9604942
```

### Carabids in pitfall traps

```
carabid.weed.pred=glmmTMB(total_weedPred_carabids~Treatment*cover_crop*Seasonality*Year*Field+(1|Block), family="genpois", ziformula=~1, data=subset(weed.seed.pred.data))
```

```
## dropping columns from rank-deficient conditional model: Treatment2IPM:cover_cropfallow:SeasonalityLate:Year2019:FieldS, Treatment3PPM:cover_cropfallow:SeasonalityLate:Year2019:FieldS
```

```
#summary(carabid.weed.pred)
#Anova(carabid.weed.pred,type=3)

simres <- simulateResiduals(carabid.weed.pred)
plot(simres)
```

Model that actually runs! - Plot\_ID estimates too small to estimate,
causing issues with null model .

```
carabid.total.selected=glmmTMB(total_weedPred_carabids~Treatment+cover_crop+Seasonality*Year*Field+(1|Block:Field)+(1|Plot:Field), family="nbinom2", ziformula=~1, data=subset(weed.seed.pred.data))
summaryOutput <- summary(carabid.total.selected)
anovaOutput <- Anova(carabid.total.selected,type=3)
r2Output <- r2_nakagawa(carabid.total.selected)

simres <- simulateResiduals(carabid.total.selected)
plot(simres)
```

```
# test the fit of fixed factors 
carabid.total.null <- glmmTMB(total_weedPred_carabids~1+(1|Block:Field)+(1|Plot:Field), family="nbinom2", ziformula=~1, data=subset(weed.seed.pred.data))
model_null_comp <- anova(carabid.total.selected,carabid.total.null)
```

ANOVA Table reporting the effects of insecticide treatment and cover
crop on carabid activity

Statistical results for Carabid beetle activity-density

| Model:  total\_weedPred\_carabids ~ Treatment + cover\_crop + Seasonality \* Year \* Field + (1 | Block:Field) + (1 | Plot:Field) | | | |
| --- | --- | --- | --- |
| N = 395 | | | |
| Model significance:   Chisq = 241.42 , df = 14 , P = 0 | | | |
| Distribution used: nbinom2 | | | |
| Conditional Nakagawa R2 (including random effects): 0.88 | | Marginal Nakagawa R2 (fixed effects only): 0.8 | |
| Fixed effect term | Chisq | Df | Pr(>Chisq) |
| (Intercept) | 0.290 | 1 | 0.591 |
| Treatment | 0.441 | 2 | 0.802 |
| cover\_crop | 9.948 | 1 | 0.002 |
| Seasonality | 24.223 | 1 | 0.000 |
| Year | 12.091 | 2 | 0.002 |
| Field | 0.834 | 1 | 0.361 |
| Seasonality:Year | 0.377 | 2 | 0.828 |
| Seasonality:Field | 9.473 | 1 | 0.002 |
| Year:Field | 25.296 | 2 | 0.000 |
| Seasonality:Year:Field | 15.010 | 2 | 0.001 |
|  |  |  |  |
| --- | --- | --- | --- |
| Block:Field - var = 0.107   Plot:Field - var = 0.026 | | | |

```
## $emmeans
##  cover_crop response    SE  df asymp.LCL asymp.UCL
##  cover          1.33 0.226 Inf      0.95      1.85
##  fallow         1.95 0.325 Inf      1.41      2.70
## 
## Results are averaged over the levels of: Treatment, Seasonality, Year, Field 
## Confidence level used: 0.95 
## Intervals are back-transformed from the log scale 
## 
## $contrasts
##  contrast       ratio     SE  df null z.ratio p.value
##  cover / fallow  0.68 0.0831 Inf    1  -3.154  0.0016
## 
## Results are averaged over the levels of: Treatment, Seasonality, Year, Field 
## Tests are performed on the log scale
```

```
## Warning: Removed 37 rows containing non-finite outside the scale range
## (`stat_boxplot()`).
```

### Ants in pitfalls

```
ants.weed.pred=glmmTMB(ants~Treatment*cover_crop*Seasonality*Year*Field*cover_crop+(1|Block)+(1|Plot)+(1|Plot_ID), family="nbinom2", data=subset(weed.seed.pred.data))
```

```
## dropping columns from rank-deficient conditional model: Treatment2IPM:cover_cropfallow:SeasonalityLate:Year2019:FieldS, Treatment3PPM:cover_cropfallow:SeasonalityLate:Year2019:FieldS
```

```
#summary(ants.weed.pred)
#Anova(ants.weed.pred,type=3)
simres <- simulateResiduals(ants.weed.pred)
plot(simres)
```

```
ants.total.selected=glmmTMB(ants~Treatment+cover_crop+Seasonality*Year+Year*Field+(1|Plot_ID:Field)+(1|Block:Field)+(1|Plot:Field), family="nbinom2", data=subset(weed.seed.pred.data))
summaryOutput <- summary(ants.total.selected)
anovaOutput <- Anova(ants.total.selected,type=3)
r2Output <- r2_nakagawa(ants.total.selected)

simres <- simulateResiduals(ants.total.selected)
plot(simres)
```

```
emmeans(ants.total.selected,pairwise~Seasonality|Field:Year,type="response")
```

```
## $emmeans
## Field = N, Year = 2017:
##  Seasonality response    SE  df asymp.LCL asymp.UCL
##  Early          21.37 2.961 Inf     16.28     28.03
##  Late            5.65 0.826 Inf      4.24      7.52
## 
## Field = S, Year = 2017:
##  Seasonality response    SE  df asymp.LCL asymp.UCL
##  Early           9.46 1.350 Inf      7.15     12.51
##  Late            2.50 0.400 Inf      1.83      3.42
## 
## Field = N, Year = 2018:
##  Seasonality response    SE  df asymp.LCL asymp.UCL
##  Early          16.82 2.308 Inf     12.86     22.01
##  Late            4.01 0.603 Inf      2.98      5.38
## 
## Field = S, Year = 2018:
##  Seasonality response    SE  df asymp.LCL asymp.UCL
##  Early          17.52 2.460 Inf     13.30     23.07
##  Late            4.17 0.617 Inf      3.12      5.57
## 
## Field = N, Year = 2019:
##  Seasonality response    SE  df asymp.LCL asymp.UCL
##  Early           6.70 1.006 Inf      4.99      8.99
##  Late            4.23 0.664 Inf      3.11      5.75
## 
## Field = S, Year = 2019:
##  Seasonality response    SE  df asymp.LCL asymp.UCL
##  Early          20.93 3.000 Inf     15.80     27.72
##  Late           13.21 2.596 Inf      8.98     19.41
## 
## Results are averaged over the levels of: Treatment, cover_crop 
## Confidence level used: 0.95 
## Intervals are back-transformed from the log scale 
## 
## $contrasts
## Field = N, Year = 2017:
##  contrast     ratio    SE  df null z.ratio p.value
##  Early / Late  3.78 0.486 Inf    1  10.358  <.0001
## 
## Field = S, Year = 2017:
##  contrast     ratio    SE  df null z.ratio p.value
##  Early / Late  3.78 0.486 Inf    1  10.358  <.0001
## 
## Field = N, Year = 2018:
##  contrast     ratio    SE  df null z.ratio p.value
##  Early / Late  4.20 0.527 Inf    1  11.439  <.0001
## 
## Field = S, Year = 2018:
##  contrast     ratio    SE  df null z.ratio p.value
##  Early / Late  4.20 0.527 Inf    1  11.439  <.0001
## 
## Field = N, Year = 2019:
##  contrast     ratio    SE  df null z.ratio p.value
##  Early / Late  1.58 0.246 Inf    1   2.964  0.0030
## 
## Field = S, Year = 2019:
##  contrast     ratio    SE  df null z.ratio p.value
##  Early / Late  1.58 0.246 Inf    1   2.964  0.0030
## 
## Results are averaged over the levels of: Treatment, cover_crop 
## Tests are performed on the log scale
```

```
# test the fit of fixed factors 
ants.total.null <- glmmTMB(ants~1+(1|Plot_ID:Field)+(1|Block:Field)+(1|Plot:Field), family="nbinom2", data=subset(weed.seed.pred.data))
model_null_comp <- anova(ants.total.selected,ants.total.null)
```

ANOVA Table reporting the effects of insecticide treatment and cover
crop on Ants

Statistical results for Ant activity-density

| Model:  ants ~ Treatment + cover\_crop + Seasonality \* Year + Year \* Field + (1 | Plot\_ID:Field) + (1 | Block:Field) + (1 | Plot:Field) | | | |
| --- | --- | --- | --- |
| N = 395 | | | |
| Model significance:   Chisq = 256.07 , df = 11 , P = 0 | | | |
| Distribution used: nbinom2 | | | |
| Conditional Nakagawa R2 (including random effects): 0.63 | | Marginal Nakagawa R2 (fixed effects only): 0.52 | |
| Fixed effect term | Chisq | Df | Pr(>Chisq) |
| (Intercept) | 378.376 | 1 | 0.000 |
| Treatment | 2.581 | 2 | 0.275 |
| cover\_crop | 0.198 | 1 | 0.656 |
| Seasonality | 107.279 | 1 | 0.000 |
| Year | 62.376 | 2 | 0.000 |
| Field | 19.138 | 1 | 0.000 |
| Seasonality:Year | 26.889 | 2 | 0.000 |
| Year:Field | 101.595 | 2 | 0.000 |
|  |  |  |  |
| --- | --- | --- | --- |
| Estimate of Random effects   Plot\_ID:Field - var = 0.071   Block:Field - var = 0.032   Plot:Field - var = 0.011 | | | |

```
## $emmeans
## Seasonality = Early:
##  cover_crop response    SE  df asymp.LCL asymp.UCL
##  cover         13.94 1.336 Inf     11.55     16.82
##  fallow        14.56 1.392 Inf     12.07     17.56
## 
## Seasonality = Late:
##  cover_crop response    SE  df asymp.LCL asymp.UCL
##  cover          4.76 0.500 Inf      3.87      5.85
##  fallow         4.97 0.524 Inf      4.04      6.11
## 
## Results are averaged over the levels of: Treatment, Year, Field 
## Confidence level used: 0.95 
## Intervals are back-transformed from the log scale 
## 
## $contrasts
## Seasonality = Early:
##  contrast       ratio     SE  df null z.ratio p.value
##  cover / fallow 0.958 0.0928 Inf    1  -0.445  0.6562
## 
## Seasonality = Late:
##  contrast       ratio     SE  df null z.ratio p.value
##  cover / fallow 0.958 0.0928 Inf    1  -0.445  0.6562
## 
## Results are averaged over the levels of: Treatment, Year, Field 
## Tests are performed on the log scale
```

```
## Warning: Removed 37 rows containing non-finite outside the scale range
## (`stat_boxplot()`).
```
