# Supplementary material for: Insecticides may facilitate the escape of weeds from biological control
Source: PeerJ. 2025 Jan 13;13:e18597. doi: 10.7717/peerj.18597 (PMC11737330; doi:10.7717/peerj.18597)
Supplement: Supplemental Information 10 — Weed seed predation measured using sentinel pigweed and foxtail seeds. This code and output used in Figure 7. [file peerj-13-18597-s010.html]

Weed Seed Predation Rowen 2024


# Weed Seed Predation Rowen 2024

#### Elizabeth Rowen

#### 5/10/2024

# Context for this document

This document includes the code for Rowen et al. submitted to Field
Crops Research in 2024 titled ” Insecticides may facilitate the escape
of weeds from biological control”

**This includes the code and statistical output for the weed
seed predator data. The code covers Results section 3.5, & Fig
7.**

**Abstract** 1. CONTEXT: Preventative pesticide seed
treatments (hereafter preventative pest management or PPM) are common in
large acreage row crops such as corn and soybean, and often include
active ingredients of both fungicides and neonicotinoid insecticides.
While PPM is intended to protect crops from soil-borne pathogens and
early season insect pests, these seed treatments may have detrimental
effects on biological control of weed seeds by insects.  
2. RESEARCH QUESTION: Here, in two 3-year corn-soy rotations in
Pennsylvania USA, we investigated a PPM approach to insect management
compared to an integrated pest management approach (IPM) and a “no
(insect) pest management” (NPM) control. This was crossed with a grass
cover crop treatment to see if this conservation practice can help
recover some of the ecosystem services affected by chemical pest
management practices. We hypothesized that PPM and IPM approaches would
release weed seeds from biological control by insects, resulting in
higher weed pressure, but cover crops would increase biological
control.  
3. METHODS: We measured the effect of these treatments on granivorous
insect activity-density, weed-seed predation, the weed-seed bank, and
mid-season weed biomass.  
4. RESULTS: We found that, contrary to our hypothesis, planting a cover
crop decreased carabid activity-density, but did not have consistent
differences in weed-seed predation. Pest management and cover crop
treatments also had inconsistent effects on the weed-seed bank and
mid-season weed biomass, but insecticide use did affect the biomass of
glyphosate-resistant marestail (*Erigeron canadensis* L.). At the
end of the trial, glyphosate-resistant marestail was more abundant in
IPM and PPM plots without a cover crop.  
5. IMPLICATIONS: Our results suggest that reducing insecticide use may
be important when combating herbicide-resistant weeds. As the adoption
conservation agriculture, including the use of no-till and cover crops,
grows, so does the use of neonicotinoid seed treatments and herbicides.
Planting cover crops and/or avoiding the use of insecticides may combat
these problematic weeds.;

### Set-up

Import libraries

```
# importing and formatting data 
library(readxl)
library(plyr)
library(flextable)
library(reshape2)
library(tidyr)
library(data.table)

# creating figures 

library(ggplot2)
library(patchwork)
# Running models 
library(car)
library(glmmTMB)
library(DHARMa)
library(vegan)
library(pscl)
#library(VGAM)
library(performance)
library(corrplot)
library(emmeans)
```

Set up colors for graphs

```
colors = c("seagreen","darkseagreen4","darkgoldenrod4", "darkgoldenrod3","tomato4","tomato3") 
colors.treat = c("seagreen","darkgoldenrod3","tomato3") # only for insecticide treatments 
colors.2017 = c("#085C3D","seagreen","#FC9078","#FFE2DB") # for 2017 when IPM had not yet been implemented 
# c("seagreen","darkseagreen4","tomato3","tomato4")
colors.cc <- c("darkgrey","antiquewhite")
```

Import and create binomial data

```
sentinel.seeds.data = read_excel('~/Dropbox/1PhD/Smith & Wickings Field Experiments (AKA smickings)/Manuscripts in progress/Weeds/PeerJ/Revision/To upload/Data&Stats/5_SentinelWeedSeeds_Apr2021.xlsx',sheet='data')

# format treatment,cover crop for each plot ID
sentinel.seeds.data=mutate(sentinel.seeds.data,Treatment=ifelse(Plot_ID=='105N' | Plot_ID=='106N'| Plot_ID=='203N'| Plot_ID=='204N'|Plot_ID=='305N'| Plot_ID=='306N'| 
                                                                  Plot_ID=='401N'|Plot_ID=='402N'| Plot_ID=='501N'| Plot_ID=='504N'| Plot_ID=='602N'| Plot_ID=='603N'| 
                                                                  Plot_ID=='102S'| Plot_ID=='106S'| Plot_ID=='201S'| Plot_ID=='204S'| Plot_ID=='303S'| Plot_ID=='305S'| 
                                                                  Plot_ID=='402S'| Plot_ID=='406S'| Plot_ID=='501S'| Plot_ID=='504S'| Plot_ID=='603S'| Plot_ID=='605S','1NPM', 
                                                                ifelse(Plot_ID=='101N' | Plot_ID=='104N'| Plot_ID=='202N'| Plot_ID=='205N'| Plot_ID=='301N'| Plot_ID=='303N'| 
                                                                         Plot_ID=='405N'| Plot_ID=='406N'| Plot_ID=='502N'| Plot_ID=='503N'| Plot_ID=='604N'| Plot_ID=='606N'| 
                                                                         Plot_ID=='104S'| Plot_ID=='105S' | Plot_ID=='202S'| Plot_ID=='203S'| Plot_ID=='301S'| 
                                                                         Plot_ID=='306S'| Plot_ID=='404S'| Plot_ID=='405S'| Plot_ID=='502S'| Plot_ID=='503S'| Plot_ID=='601S'| 
                                                                         Plot_ID=='606S','2IPM','3PPM')))


sentinel.seeds.data=mutate(sentinel.seeds.data,cover_crop=ifelse(Plot_ID=='101N' | Plot_ID=='102N'| Plot_ID=='106N'| Plot_ID=='203N'|Plot_ID=='205N'| Plot_ID=='206N'|
                                                                   Plot_ID=='303N'| Plot_ID=='304N'| Plot_ID=='305N'| Plot_ID=='401N'| Plot_ID=='403N'| Plot_ID=='406N'| 
                                                                   Plot_ID=='502N'| Plot_ID=='504N'|Plot_ID=='505N'| Plot_ID=='601N'| Plot_ID=='602N'| Plot_ID=='604N'| 
                                                                   Plot_ID=='102S'| Plot_ID=='103S'| Plot_ID=='105S'| Plot_ID=='202S'| Plot_ID=='204S'| Plot_ID=='206S'| 
                                                                   Plot_ID=='303S'| Plot_ID=='304S'| Plot_ID=='306S'| Plot_ID=='401S'|  Plot_ID=='404S'| Plot_ID=='406S'| 
                                                                   Plot_ID=='501S'| Plot_ID=='503S'| Plot_ID=='505S'| Plot_ID=='601S'| Plot_ID=='602S'|Plot_ID=='605S','cover', 'fallow'))
sentinel.seeds.data=mutate(sentinel.seeds.data,Treated=ifelse(Year == '2017' &Treatment=='2IPM'|Treatment== '1NPM','1NPM',Treatment))

# format sentinel weed seeds so that each seed has an outcome 

# Weed seed predation 
sentinel.seeds.data$Year=as.factor(sentinel.seeds.data$Year)
sentinel.seeds.data$FT_eaten=20-sentinel.seeds.data$FT
sentinel.seeds.data$PW_eaten=30-sentinel.seeds.data$PW
#sentinel.seeds.data = subset(sentinel.seeds.data,FT_eaten>=0 & PW_eaten>=0)# use only positive values
sentinel.seeds.data$total_eaten=sentinel.seeds.data$FT_eaten+sentinel.seeds.data$PW_eaten
#sentinel.seeds.data1=melt(sentinel.seeds.data,id=c('Year','Date','Month','Time','Field','Block','Row','Plot_ID','row','Notes','Treatment','cover_crop','Treated'))


sentinel.seeds.data.small = sentinel.seeds.data[c("Year","Time","Field","Block","Row","Plot_ID","row","Treatment","cover_crop","FT_eaten","PW_eaten","total_eaten")]
sentinel.seeds.data.small =as.data.table(sentinel.seeds.data.small)
sentinel.seeds.data.small$PW_remaining=30-sentinel.seeds.data.small$PW_eaten
sentinel.seeds.data.small$FT_remaining=20-sentinel.seeds.data.small$FT_eaten
sentinel.seeds.data.small$total_remaining=50-sentinel.seeds.data.small$total_eaten
sentinel.seeds.data.small <- sentinel.seeds.data.small[PW_eaten>0&FT_eaten>0&total_eaten>0]

sentinel.seeds.data.melt = data.table(melt(sentinel.seeds.data.small,id.vars=c("Year","Time","Field","Block","Row","Plot_ID","row","Treatment","cover_crop")))
sentinel.seeds.data.melt <- sentinel.seeds.data.melt[sentinel.seeds.data.melt$value!="NA",]

# new data table with an outcome for each seed 

new.table = data.table(NULL)

for(i in 1:dim(sentinel.seeds.data.melt)[1]){
  ref.row=sentinel.seeds.data.melt[i,]
  #print(i)
  #print(ref.row)
  #x=c(x,ref.row$value)
  for(j in 1:ref.row$value){
    new.table=rbind(new.table,ref.row)
  }
}

# make 2 columns, one with FT and one with PW 
new.table=mutate(new.table,seed.outcome=ifelse(value>=1&variable=="FT_eaten"|value>=1&variable=="PW_eaten"|value>=1&variable=="total_eaten",1,0))
sentinel.seed.data.binom=mutate(new.table,seeds=ifelse(variable=="FT_eaten"|variable=="FT_remaining",'FT',ifelse(variable=="PW_eaten"|variable=="PW_remaining","PW","total")))
```

### Predatation stats for each field year (Table 2)

```
predation.summary <- ddply(sentinel.seeds.data, .(Year, Field,Time), summarize, mean.beetle.count = mean (total_eaten,na.rm=T), N = sum(!is.na(total_eaten)),sd=sd(total_eaten,na.rm=T),se=sd/sqrt(N));predation.summary
```

```
##    Year Field   Time mean.beetle.count   N        sd        se
## 1  2017     N 1Early          5.240741 108  8.715515 0.8386508
## 2  2017     N   2Mid          6.268519 108 10.178414 0.9794184
## 3  2017     N  3Late          2.542857 105  3.700386 0.3611207
## 4  2017     S 1Early          3.148148 108  5.849858 0.5629028
## 5  2017     S   2Mid         12.129630 108 14.037395 1.3507490
## 6  2017     S  3Late         27.018519 108 18.720762 1.8014062
## 7  2018     N 1Early         14.145631 103  7.432583 0.7323542
## 8  2018     N   2Mid         14.485437 103 13.348176 1.3152349
## 9  2018     N  3Late         35.205607 107 15.241910 1.4734910
## 10 2018     S 1Early         11.990741 108 10.529438 1.0131957
## 11 2018     S   2Mid         34.621359 103 15.477445 1.5250380
## 12 2018     S  3Late         37.504673 107 18.098459 1.7496441
## 13 2019     N 1Early          8.175926 108  5.987259 0.5761242
## 14 2019     N   2Mid          7.308411 107  7.433239 0.7185983
## 15 2019     N  3Late         17.490385 104 12.127955 1.1892438
## 16 2019     S 1Early          3.064815 108  4.965814 0.4778357
## 17 2019     S   2Mid         15.808081  99 14.160650 1.4231989
## 18 2019     S  3Late         22.974684  79 16.420730 1.8474765
```

### Binomial seed predation

```
###      use binomial model with data for each seed 

weed.tot.pred=glmmTMB(seed.outcome~Treatment*cover_crop*Year*Field*Time+(1|Plot_ID:Field/Time/row)+(1|Block:Field/Time)+(1|Row:Field/Time),family=binomial, data=subset(sentinel.seed.data.binom,seeds=="total"))
summaryOutput <- summary(weed.tot.pred)
anovaOutput <- Anova(weed.tot.pred)
r2Output <- r2_nakagawa(weed.tot.pred)
```

```
## Warning: Can't compute random effect variances. Some variance components equal
##   zero. Your model may suffer from singularity (see `?lme4::isSingular`
##   and `?performance::check_singularity`).
##   Solution: Respecify random structure! You may also decrease the
##   `tolerance` level to enforce the calculation of random effect variances.
```

```
## Random effect variances not available. Returned R2 does not account for random effects.
```

```
model_N <- summaryOutput$nobs


simres <- simulateResiduals(weed.tot.pred)
plot(simres)
```

```
# compare to null model
weed.tot.pred.null=glmmTMB(seed.outcome~1+(1|Plot_ID:Field/Time/row)+(1|Block:Field/Time)+(1|Row:Field/Time),family=binomial,
                      data=subset(sentinel.seed.data.binom,seeds=="total"))
model_null_comp <-anova(weed.tot.pred,weed.tot.pred.null)
```

ANOVA Table reporting the effects of insecticide treatment and cover
crop on weed seed predation rate

Statistical results for weed seed predation

| Model:  seed.outcome ~ Treatment \* cover\_crop \* Year \* Field \* Time + (1 | Plot\_ID:Field/Time/row) + (1 | Block:Field/Time) + (1 | Row:Field/Time) | | | |
| --- | --- | --- | --- |
| N = 63292 | | | |
| Model significance:   Chisq = 6677.23 , df = 107 , P = 0 | | | |
| Distribution used: binomial | | | |
| Conditional Nakagawa R2 (including random effects): NA | | Marginal Nakagawa R2 (fixed effects only): 0.35 | |
| Fixed effect term | Chisq | Df | Pr(>Chisq) |
| Treatment | 8.052 | 2 | 0.018 |
| cover\_crop | 0.121 | 1 | 0.728 |
| Year | 3,500.459 | 2 | 0.000 |
| Field | 20.301 | 1 | 0.000 |
| Time | 205.767 | 2 | 0.000 |
| Treatment:cover\_crop | 0.498 | 2 | 0.780 |
| Treatment:Year | 55.006 | 4 | 0.000 |
| cover\_crop:Year | 25.379 | 2 | 0.000 |
| Treatment:Field | 4.288 | 2 | 0.117 |
| cover\_crop:Field | 2.289 | 1 | 0.130 |
| Year:Field | 76.440 | 2 | 0.000 |
| Treatment:Time | 7.702 | 4 | 0.103 |
| cover\_crop:Time | 0.223 | 2 | 0.894 |
| Year:Time | 302.379 | 4 | 0.000 |
| Field:Time | 49.382 | 2 | 0.000 |
| Treatment:cover\_crop:Year | 32.176 | 4 | 0.000 |
| Treatment:cover\_crop:Field | 1.586 | 2 | 0.453 |
| Treatment:Year:Field | 44.853 | 4 | 0.000 |
| cover\_crop:Year:Field | 13.788 | 2 | 0.001 |
| Treatment:cover\_crop:Time | 2.652 | 4 | 0.618 |
| Treatment:Year:Time | 51.798 | 8 | 0.000 |
| cover\_crop:Year:Time | 35.640 | 4 | 0.000 |
| Treatment:Field:Time | 7.503 | 4 | 0.112 |
| cover\_crop:Field:Time | 1.816 | 2 | 0.403 |
| Year:Field:Time | 533.142 | 4 | 0.000 |
| Treatment:cover\_crop:Year:Field | 17.906 | 4 | 0.001 |
| Treatment:cover\_crop:Year:Time | 209.763 | 8 | 0.000 |
| Treatment:cover\_crop:Field:Time | 6.253 | 4 | 0.181 |
| Treatment:Year:Field:Time | 220.357 | 8 | 0.000 |
| cover\_crop:Year:Field:Time | 18.617 | 4 | 0.001 |
| Treatment:cover\_crop:Year:Field:Time | 38.174 | 8 | 0.000 |
|  |  |  |  |
| --- | --- | --- | --- |
| Estimate of Random effects   row:Time:Field:Plot\_ID - var = 0.86   Time:Field:Plot\_ID - var = 0   Field:Plot\_ID - var = 0   Time:Field:Block - var = 0.068   Field:Block - var = 0.013   Time:Field:Row - var = 0   Field:Row - var = 0.009 | | | |

Figure 7. Estimated marginal mean probability (95% confidence
intervals [CIs]) of weed seeds eaten at each sampling event. Means for
treatments with cover crops indicated with a solid line, means without
cover crop indicated by a dashed line. Groups of means that share
capitalized letters are significantly different among sampling points
within a given site-year, where GLM indicated seasonality had a
significant effect by year/field slicing. Plot means that share lower
case letters are significantly different where GLM indicated CC x PM had
a significant effect by season/year/field slicing.

```
x=emmeans(weed.tot.pred, ~Treatment:cover_crop:Year:Field:Time,type="response")
weed.pred.table = as.data.frame(x)

tmp=melt(weed.pred.table, id=c("Field","cover_crop","Treatment","Year",'Time'))
```

```
## Warning: The melt generic in data.table has been passed a summary_emm and will
## attempt to redirect to the relevant reshape2 method; please note that reshape2
## is superseded and is no longer actively developed, and this redirection is now
## deprecated. To continue using melt methods from reshape2 while both libraries
## are attached, e.g. melt.list, you can prepend the namespace, i.e.
## reshape2::melt(weed.pred.table). In the next version, this warning will become
## an error.
```

```
tmp$value=round(tmp$value,2)
tmp2=dcast(tmp,Field+Treatment+cover_crop+Year+Time~variable)
```

```
## Warning: The dcast generic in data.table has been passed a data.frame and will
## attempt to redirect to the relevant reshape2 method; please note that reshape2
## is superseded and is no longer actively developed, and this redirection is now
## deprecated. Please do this redirection yourself like reshape2::dcast(tmp). In
## the next version, this warning will become an error.
```

```
tmp2$CI=paste(tmp2$asymp.LCL,"\U2012",tmp2$asymp.UCL)
tmp3=melt(tmp2, id=c("Field","cover_crop","Treatment","Year","Time"))
```

```
## Warning: The melt generic in data.table has been passed a data.frame and will
## attempt to redirect to the relevant reshape2 method; please note that reshape2
## is superseded and is no longer actively developed, and this redirection is now
## deprecated. To continue using melt methods from reshape2 while both libraries
## are attached, e.g. melt.list, you can prepend the namespace, i.e.
## reshape2::melt(tmp2). In the next version, this warning will become an error.
```

```
weed.pred.table1=dcast(subset(tmp3,variable=="prob"|variable=="asymp.LCL"|variable=="asymp.UCL"),Time+Field+Treatment+cover_crop+Year~variable)
```

```
## Warning: c("The dcast generic in data.table has been passed a data.frame and will attempt to redirect to the relevant reshape2 method; please note that reshape2 is superseded and is no longer actively developed, and this redirection is now deprecated. Please do this redirection yourself like reshape2::dcast(subset(tmp3, variable == \"prob\" | variable == \"asymp.LCL\" | variable == ). In the next version, this warning will become an error.", "The dcast generic in data.table has been passed a data.frame and will attempt to redirect to the relevant reshape2 method; please note that reshape2 is superseded and is no longer actively developed, and this redirection is now deprecated. Please do this redirection yourself like reshape2::dcast(    \"asymp.UCL\")). In the next version, this warning will become an error."
## )
```

```
weed.pred.table1$prob <- as.numeric(weed.pred.table1$prob)
weed.pred.table1$asymp.LCL<- as.numeric(weed.pred.table1$asymp.LCL)
weed.pred.table1$asymp.UCL<- as.numeric(weed.pred.table1$asymp.UCL)

weed.pred.table1$cover_crop=ordered(weed.pred.table1$cover_crop, levels = c("fallow","cover"))

# plot change over the season 
    
    p=ggplot(data=weed.pred.table1,aes(y=as.numeric(prob),x=Time,shape=paste(Treatment,'\n',cover_crop),
                                        fill=paste(Treatment,'\n',cover_crop)))
    
    p=p+geom_errorbar(color='darkgrey',position=position_dodge(0.5),
                      aes(y=as.numeric(prob),x=Time,ymin=as.numeric(asymp.LCL), ymax=as.numeric(asymp.UCL)),
                      width=0.2)
    p=p+geom_point(size=3,color="black",position=position_dodge(width=0.5))
    p=p+geom_line(data=weed.pred.table1,position=position_dodge(width=0.5),aes(y=as.numeric(prob),x=Time,group=paste(Treatment,'\n',cover_crop),linetype=cover_crop))
    p=p+theme_classic()+scale_color_manual(values=colors)+ scale_fill_manual(values=colors)+
      scale_shape_manual(values=c(21,22,23,24,25,21))# + ylim(0,40)+xlab('')
    p=p+ylab("Total weed seed predation")+facet_grid(Field~Year)+theme(legend.position="none")
    p.weedPred = p
    p.weedPred
```

```
    #ggsave(p.weedPred,file="~/Dropbox/1PhD/Smith & Wickings Field Experiments (AKA smickings)/Manuscripts in progress/Weeds/Total_seed_predationLine.eps",width=15,height=7.5,units='in')
```
